# The relationship between the therapeutic alliance in psychotherapy and suicidal experiences: A systematic review

**DOI:** 10.1002/cpp.2726

**Published:** 2022-02-25

**Authors:** Charlotte Huggett, Patricia Gooding, Gillian Haddock, Jody Quigley, Daniel Pratt

**Affiliations:** ^1^ Division of Psychology and Mental Health, School of Health Sciences, Manchester Academic Health Science Centre University of Manchester Manchester UK; ^2^ Greater Manchester Mental Health NHS Foundation Trust, Manchester Academic Health Science Centre Manchester UK; ^3^ Pennine Care NHS Foundation Trust Ashton‐under‐Lyne UK

**Keywords:** psychotherapy, suicide, systematic review, Therapeutic alliance

## Abstract

It is well established that there is a fundamental need to develop a robust therapeutic alliance to achieve positive outcomes in psychotherapy. However, little is known as to how this applies to psychotherapies which reduce suicidal experiences. The current narrative review summarizes the literature which investigates the relationship between the therapeutic alliance in psychotherapy and a range of suicidal experiences prior to, during and following psychotherapy. Systematic searches of MEDLINE, PsycINFO, Web of Science, EMBASE and British Nursing Index were conducted. The search returned 6472 studies, of which 19 studies were eligible for the present review. Findings failed to demonstrate a clear link between suicidal experiences prior to or during psychotherapy and the subsequent development and maintenance of the therapeutic alliance during psychotherapy. However, a robust therapeutic alliance reported early on in psychotherapy was related to a subsequent reduction in suicidal ideation and attempts. Study heterogeneity, varied sample sizes and inconsistent reporting may limit the generalizability of review findings. Several recommendations are made for future psychotherapy research studies. Training and supervision of therapists should not only highlight the importance of developing and maintaining the therapeutic alliance in psychotherapy when working with people with suicidal experiences but also attune to client perceptions of relationships and concerns about discussing suicidal experiences during therapy.

**Key Practitioner Message**

- This is the first review to investigate the relationship between the therapeutic alliance in psychotherapy and suicidal experiences *pre‐*therapy, *during* therapy and *after* therapy.
- There is no clear link between suicidal experiences prior to psychotherapy and the strength of the therapeutic alliance.
- A robust, client‐viewed, therapeutic alliance established early in psychotherapy is related to reduced future suicidal experiences.
- Training and supervision of therapists should highlight the importance of, and, key considerations, when developing and maintaining a therapeutic alliance with people with suicidal experiences.
- Practitioners involved in psychotherapy trials with suicidal experience outcomes should routinely measure the therapeutic alliance and assess the relationship between alliance and suicidal experiences.

## 1 INTRODUCTION

Suicidal ideation, attempts and deaths by suicide are a major global health concern and a public health priority. Estimates show that in 2018, 14.8 in every 100,000 people in the United States (Centers for Disease Control and Prevention, 2020) and 11.2 in every 100,000 people in the United Kingdom (Office for National Statistics, 2019) died by suicide. The risk of death by suicide is higher in people with mental health diagnoses, such as borderline personality disorder (45.1%) and depression (19.7%), than within the general population (Chesney et al., 2014). Suicidal ideation and suicide plans have also been described as key predictors of suicide attempts and suicide deaths (Bertelsen et al., 2007; O'Connor & Kirtley, 2018). Furthermore, male gender, fewer years spent in education, a history of physical and repeated sexual abuse, unemployment and homelessness increase the risk of suicidal experiences (Nock & Kessler, 2006; Schneider et al., 2011; Windfuhr & Kapur, 2011).

Evidence‐based psychotherapies, which are grounded in contemporary models of the psychological mechanisms underpinning suicide, have been developed to target suicidal thoughts and behaviour (Johnson et al., 2008; Joiner & Silva, 2012; O'Connor & Kirtley, 2018; Williams, 1997). There is evidence from two meta‐analytic reviews to suggest that psychotherapies such as cognitive therapy (CT), cognitive behavioural therapy (CBT), dialectical behaviour therapy (DBT), mentalization‐based treatment and interpersonal psychotherapy reduce suicidal behaviour (Calati & Courtet, 2016; Tarrier et al., 2008).

For the purpose of this review, a definition of psychotherapy was based on that of Beutler (2009): the consideration of client and therapist factors, development of the client‐therapist alliance and implementation of therapeutic techniques which aim to facilitate beneficial change for people with mental health problems. One aspect of psychotherapy, which has gained significant attention, is the therapeutic alliance. In broad terms, the therapeutic alliance captures perceptions of the evolving working relationship between a client and therapist in a wide range of clinical interactions, including psychological talking therapies. The alliance, as perceived by both therapists and clients, is recognized as pivotal to a positive outcome from psychotherapy (Flückiger et al., 2018). This is a finding which traverses a variety of therapeutic modalities and a diverse range of mental health problems (Flückiger et al., 2018).

Despite the abundance of research indicating a relationship between the therapeutic alliance and therapeutic outcomes, a query remains over whether the therapeutic alliance is, indeed, a predictor of outcome alone, a development which resulted from expectations of psychotherapy and/or a facilitator of effective psychotherapy (Horvath, 2006; Zilcha‐Mano, 2017). A possible barrier to addressing such a query within the current literature is that the majority of measures of therapeutic alliance are captured at one time point during psychotherapy. This limits insight into the alliance–outcome relationship at other time points during therapy (Zilcha‐Mano, 2017). A new model has been proposed by Zilcha‐Mano (2017) for understanding the possible therapeutic nature of the alliance, which posited that client ‘trait‐like’ (e.g. patterns of relating, expectations of relationships and appraisals of themselves and interactions with others) and alliance ‘state‐like’ (e.g. ‘in‐the‐moment’ dynamic and therapeutic nature of the alliance itself) components contribute to therapeutic change. That said, the use of the term ‘trait’ implies that the characteristics that clients bring to the therapeutic situation are unable to change, whereas a fundamental aim of therapy is to bring about change. Moreover, it appears that this model is yet to be empirically tested. Furthermore, measures of the therapeutic alliance need to be reviewed to examine whether they are sensitive to session‐by‐session therapeutic change (Zilcha‐Mano, 2017). Therefore, session‐by‐session ratings of therapeutic alliance may allow researchers to better understand if the alliance is uniquely related to therapeutic change.

Three factors in the development of the therapeutic alliance have been scrutinized, which include the effect of mental health problems on the development of the alliance, breakdowns or ruptures (Safran et al., 1990) and the effect that the alliance has on positive changes in mental health problems subsequent to therapy (DeRubeis & Feeley, 1990). That said, the pathways to perceived helpful therapy may be cyclical or non‐linear.

In terms of pre‐therapy experiences, the severity of anxiety, depression, psychosis, attachment style and number of traumatic events were not associated with client perspectives of the alliance early on in psychological therapies, such as supportive expressive psychotherapy and CBT (Gibbons et al., 2003;Reynolds et al., 2017; Shattock et al., 2018). In contrast, experience of depression pre‐therapy has been significantly related to poorer client perception of the alliance (Shattock et al., 2018). Additionally, depression and coping styles such as acceptance and seeking emotional support prior to starting therapy were significantly correlated with client perception of a stronger therapeutic alliance (Reynolds et al., 2017; Shattock et al., 2018). Despite the conflicting evidence presented within the literature, mental health problems and coping styles that pre‐existed before the start of therapy may lend to the investment in a stronger initial client–therapist bond, which could positively feed into a strengthening of the alliance in therapy.

The therapeutic alliance has been recognized as non‐linear, often fluctuating, during the course of psychotherapy. Events such as alliance ruptures (e.g. breakdown in communication and poor understanding) and the resolution of such ruptures may occur between the client and therapist (Safran et al., 1990; Safran & Muran, 2006). It is necessary for the therapist to be able to recognize when ruptures occur and to negotiate with the client ways of resolving such ruptures. It has been posited that alliance ruptures and harmful client–therapist interactions may be risk factors for adverse reactions to therapy (Parry et al., 2016). However, studies have suggested that alliance ruptures and subsequent repairs are associated with not only positive outcomes and a stronger therapeutic alliance in psychotherapy (Muran et al., 2009) but also greater improvements in mental health problems, compared to no experience of alliance ruptures (Stiles et al., 2004). This may be due to clients learning from interpersonal struggles (Safran et al., 1990; Safran & Muran, 2000). Nevertheless, it is important to monitor and address the occurrence of alliance ruptures and harmful interactions in therapy to ensure the safe delivery of therapy and mitigate against possible adverse reactions to therapy (Parry et al., 2016).

The alliance–outcome relationship is well established. Not only has a stronger client‐therapist alliance predicted positive outcomes post‐therapy (Flückiger et al., 2018), but the possible reciprocal relationship with psychological distress has been explored. Evidence pertaining to this issue largely comes from research involving those experiencing anxiety and/or depression. Early on in CBT, improvement in experiences of depression were found to be related to a more robust therapeutic alliance, but the alliance was not found to be related to subsequent improvement in experiences (DeRubeis & Feeley, 1990; Strunk et al., 2010). Moreover, a stronger therapeutic alliance developed during supportive‐expressive psychotherapy was associated with less severe experiences of depression across four time‐points. But severity of depression was not associated with the perceived strength of the therapeutic alliance at subsequent time points (Zilcha‐Mano et al., 2014). Additionally, a reciprocal temporal relationship between the therapeutic alliance and changes in severity of depression and psychological distress has been observed during the delivery of a range of psychotherapies, including, cognitive behavioural, psychodynamic and alliance‐fostering approaches (Crits‐Christoph et al., 2011; Falkenström et al., 2013). Therefore, perceptions of a positive therapeutic alliance, especially when formed during initial sessions, may lead to subsequent reductions in psychological distress early on in psychotherapy which may in turn positively reinforce an even stronger therapeutic alliance. It remains unclear how generalizable such findings are to populations experiencing other mental health problems or different types of therapy. One area for which there is relatively scant research is the effect of severe mental health problems and suicidal experiences prior to starting therapy on the client–therapy alliance.

**FIGURE 1:**
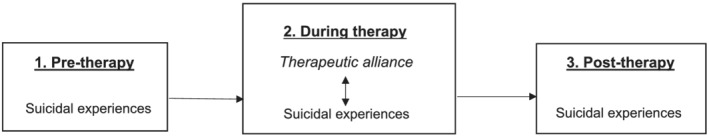
A diagram to illustrate the direction of the three types of relationship under investigation between the therapeutic alliance and suicidal experiences

Considering the significance of the therapeutic alliance and therapeutic outcome, very few suicide prevention‐focused intervention studies have examined the contribution of the therapeutic alliance upon suicidal outcome variables. One existing review has broadly explored the relationship between therapeutic alliance and suicidal ideation, self‐harm and suicide attempts in people accessing mental health services or receiving psychotherapy (Dunster‐Page et al., 2017). Findings indicated that a more robust therapeutic alliance was associated with a reduction in suicidal thoughts and instances of self‐harm, whereas there were mixed results regarding the relationship with suicide attempts (Dunster‐Page et al., 2017). Such inconsistencies could be due to lower frequency of suicide attempts and therefore less power to detect a relationship. The focus of the review was quite broad, looking at the alliance in both inpatient and outpatient mental health teams in the United States, individual care coordinators from community mental health teams in the United Kingdom, as well as psychotherapy, and the relationship between both suicide and self‐harm outcomes. Thus far, there is a gap in the evidence base, whereby the direction of the relationship between the therapeutic alliance established during psychotherapy and suicidal experiences has not yet been investigated using systematic review methods. Hence, the overarching aims of the current review were to investigate the nature of the relationship between the therapeutic alliance in psychotherapy and suicidal experiences by examining the evidence for suicidal thoughts and behaviours as (1) predictors of the alliance (i.e. suicidal experiences *pre‐therapy* influencing the therapeutic alliance), (2) correlates of the alliance (i.e. suicidal experiences related to the therapeutic alliance *at the same time point during* psychotherapy) and (3) outcomes due to the therapeutic alliance (i.e. the therapeutic alliance altering suicidal experiences *post‐therapy* [see Figure 1]). An additional aim was to assess the reliability, validity, applicability, findings and reporting of studies which are published and included in the current systematic review.

## 2 METHOD

The current systematic review was conducted and is reported in line with Preferred Reporting Items for Systematic reviews and Meta‐Analyses (PRISMA; Liberati et al., 2009) and was registered on the Prospero Centre for Reviews and Dissemination website (CRD42019138823).

### 2.1 Search strategy

The database search strategy was carried out from 1976 (MEDLINE, Embase, PsycINFO and Web of Science) or date of inception (1987; British Nursing Index) to December 2021. The search was limited to 1976 as this is predominantly when the first therapeutic alliance measures were developed (Luborsky, 1976). A restriction on English language was applied. Search terms comprised phrases relating to suicide, psychotherapy and therapeutic alliance, all separated by the Boolean operator; ‘AND’. The first search term was ‘suicid*’ to capture all studies relating to suicidal experiences such as suicidal ideation and attempts and death by suicide. The second set of search terms were those related to psychotherapy: ‘cognitiv*’ OR ‘psychotherap*’ OR ‘psycholog* therap*’ OR ‘psychosocial’ OR ‘talking therap*’ OR ‘counseling’ OR ‘counselling’ OR ‘talking treatment’ OR ‘psycholog* intervention*’. The final set of search terms were related to the therapeutic alliance: ‘alliance’ OR ‘therap* relation*’ OR ‘bond*’ OR ‘connection’ OR ‘rapport’ OR ‘collaborat*’ OR ‘therap* attachment’ OR ‘engage*’ OR ‘empath*’ OR ‘withdraw*’ OR ‘therap* delivery’ OR ‘therap* process’. Forward and backward citation chaining (Booth et al., 2013) was utilized to account for the possibility of potential peer‐reviewed articles being missed in the original search. This technique involved using the ‘finding citing articles’ feature on Ovid to identify relevant studies which cited included studies, in addition to examining reference lists for all studies included in the present review. The use of citation chaining is encouraged to ensure the review strategy is comprehensive (Booth et al., 2013).

### 2.2 Eligibility criteria

Studies were deemed eligible for inclusion if they met the following criteria: (1) written in English; (2) quantitative empirical studies; (3) published in a peer‐reviewed academic journal; (4) involved individuals of any age, gender, ethnicity and presenting mental health problem who have had suicidal experiences (i.e. suicidal ideation or attempts) in their lifetime or had died by suicide; (5) involved a psychotherapeutic intervention delivered individually or in a group at any point in time; (6) any measure of therapeutic alliance; (7) any measure of suicidal experiences (such criteria ensured that measures which may not be validated questionnaires, e.g. hospital or other records, were included); and (8) reported analyses of the relationship between the therapeutic alliance and at least one type of suicidal experience.

Studies were excluded if they met the following criteria: (1) review articles, clinical practice, position papers, treatment guidelines, grey literature and qualitative only studies; (2) intervention was solely pharmacological therapy (i.e. medicinal treatments), alternative medicinal or other treatment (i.e. homoeopathy, acupuncture, osteopathy, chiropractic, herbal medicines, aromatherapy and prescribed exercise) or self‐guided interventions, including interventions which primarily use technology (i.e. smartphone application or website where a human therapist is not conducting psychotherapy).

### 2.3 Study selection

Titles and abstracts were screened by the first author (CH). Full texts of potentially eligible papers were then examined by the first author to confirm eligibility. A random sample of 13.5% (*n* = 32) of all full texts was screened by a second independent reviewer (JQ) to determine inter‐rater reliability. Disagreements were resolved through discussion. Overall, there was 100% agreement (κ = 1). Queries regarding whether studies met with the eligibility criteria were resolved by discussed with three experienced clinical and academic psychologists (PG, GH, DP).

### 2.4 Data extraction and analysis

Data were extracted with reference to a data extraction table, which had been created and piloted by the first author, comprising study characteristics, client and therapist characteristics, modes of therapy delivery and data analysis (see Appendix A for more specific details of data extracted). For those studies that measured the therapeutic alliance and suicidal experiences but did not analyse the relationship between these two variables, the relevant data or analyses were then requested from the corresponding authors. Of 27 authors who were contacted, four provided the necessary data analysis. Corresponding authors from 17 out of 19 included studies were contacted to request missing data.

### 2.5 Quality assessment using the Critical Appraisal Skills Programme

There is no consensus as to which quality assessment tool is most suitable for use across a variety of study designs (Katrak et al., 2004). Included studies in the current review collected data using RCT and cohort designs. However, specific questions pertaining to the quality of randomization processes were not applicable to the current review question, which focused on the therapeutic alliance in psychotherapy. Additionally, the Critical Appraisal Skills Programme (CASP) (2018) checklist for cohort studies has been specifically recommended for critical appraisals of cohort studies (Rosella et al., 2016). The CASP checklists provide clinicians and researchers with a framework to assess the reliability, validity, applicability, findings and reporting of studies which are published. As the questions posed by the CASP checklist were broad, each question was tailored to the current systematic review topic to ensure the quality assessment of studies was relevant. For example, adapted questions included assessing whether measures of therapeutic alliance and suicidal experiences were reliable and valid, the therapists were systematically trained, therapist fidelity monitored and the psychotherapy was safe. Therefore, each study was quality assessed using an adapted version of the CASP (2018) checklist for specific study designs.

The first author (CH) quality assessed all included studies, of which five (26%) were also assessed by an independent second reviewer (JQ) to determine inter‐rater reliability. Four studies were selected at random based on each study design. However, one study was specifically selected to be independently quality assessed as the first author of the present review is the first author of the included paper. There was 96% agreement (κ = .92) on the CASP ratings for the five selected papers.

## 3 RESULTS

### 3.1 Search results

**FIGURE 2:**
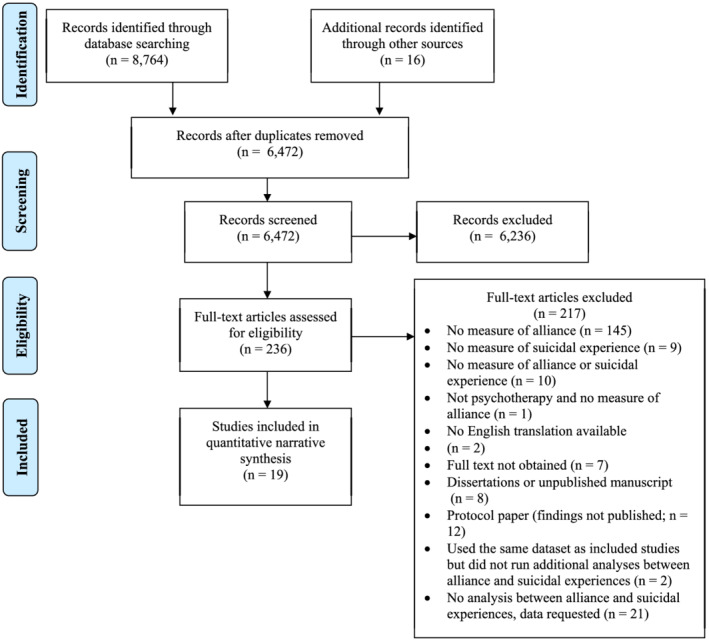
PRISMA diagram

A summary of study flow from initial database search to inclusion at full‐text level are presented in Figure 2. Notably, 23 studies measured both the therapeutic alliance and suicidal experiences but did not conduct a statistical analysis of the relationship between these variables. Nineteen studies met inclusion criteria and were included in this systematic review, of which two sets of two studies (*n* = 4) analysed data from the same pool of participants.

### 3.2 Study characteristics

As might be expected, there was considerable heterogeneity across studies with respect to geographical location, study design, settings, sample sizes, participant characteristics, types and delivery mode of therapy offered, characteristics of the therapists, measures of the therapeutic alliance, measures of suicidal experiences and study quality. Furthermore, analyses examined different directions of the relationship between the therapeutic alliance and suicidal experiences (pre‐therapy, during therapy and post‐therapy). The number of times each variable was measured and at which time points also varied considerably (e.g. baseline, during therapy at a single time point or session by session, upon therapy cessation and either once or multiple times at follow‐up time points). Additionally, some studies had low retention rates, unclear therapy or follow‐up timeframes and/or did not report or provide sufficient data (standard deviations, standard errors and confidence intervals). Due to clinical and methodological diversity, statistical heterogeneity and insufficient data, a meta‐analysis examining the relationships between the therapeutic alliance and suicidal experiences was considered inappropriate (Higgins & Green, 2011). Nine out of 19 studies were conducted in the United States, four in Canada and six in Europe.

Study details, such as design, study setting/recruitment sources, sample sizes, sample population, type of psychotherapy, therapy delivery characteristics, therapist qualifications and supervision, have been collated in Table 1. There are, however, several key points to note. For instance, most studies used a cohort/longitudinal or randomized controlled trial (RCT) design (including pilot RCTs) using opportunity sampling from the community, mental health inpatient and outpatient settings, with sample sizes ranging from 4 to 633 and follow‐up time periods between 2 weeks and a median of 4.19 years. Participants with different mental health problems were recruited across studies, but those with a diagnosis of borderline personality disorder were represented most frequently, whereas people diagnosed with eating disorders, bipolar disorder or non‐affective psychosis had the least frequent representation. The mean age of participants ranged from just under 15 to just over 48 years. Ethnicity was predominantly Caucasian or not reported. Seven out of 13 RCTs compared psychological therapy with an active control (e.g. client‐centred, non‐directive supportive family therapy, psychodynamic, eclectic or cognitive therapy). The experience of the study therapists, who came from various allied mental health professions (e.g. social work, psychology and nursing) and were either in training or had qualifications ranging from masters and PhD degrees to professional registration in clinical psychology and psychiatry, ranged from 1 to 26 years. The types of therapy offered were also diverse including cognitive, psychodynamic and eclectic approaches, delivered in one‐to‐one settings (nine studies), groups (three studies) or a mixture of group and individual work (seven studies). The number of therapy sessions ranged from 3 to 339, but most (*n* = 12) studies offered 3–20 weekly sessions lasting between 60 and 180 min.

**TABLE 1:** Included study characteristics in date order from oldest to most recent, participant age and ethnicity, details of psychotherapy delivery, format and context and therapist qualifications and supervision

	Study characteristics			Participants			Psychotherapy delivery, format and context		Therapists	
Study number and reference	Country	Design	Sample population and study setting	Therapy arm sample size	Mean age	Ethnicity and Gender	Psychotherapy type and session length	Length and setting of psychotherapy	N and qualifications	Supervision
1. Shearin and Linehan (1992)	USA	Cohort/longitudinal	People with a diagnosis of BPD and parasuicidal behaviour in the community	4	Not reported	Not reported; 100% female	**Dialectical behavioural therapy (DBT):** 60‐min individual sessions and 150‐min group skills per week	Up to 31 sessions over 7 months at an outpatient university research clinic	4 psychology and nursing graduate students	Supervision provided to ensure adherence to DBT protocol, but no further details reported
2. Turner (2000)	USA	Two‐armed RCT; active control	People with a diagnosis of BPD in the community	**Total:** 24 **DBT:** 12	**Total:** 22.00	**Total:** 79.17% Caucasian; 79.17% female	**DBT:** Individual (DBT skills sessions provided in individual sessions)^a^	Up to 84 individual sessions over 12 months at a community mental health outpatient clinic	4 therapists; background in client‐centred, psychodynamic and family systems conducted both therapies **DBT:** Trained to conduct DBT	Weekly group supervisions (one for each therapeutic modality). Reviewed therapy audio recordings to monitor treatment fidelity
				**Active control:** 12			**Active control:** Individual client‐centred therapy^a^		**Active control:** Trained to work with people diagnosed with BPD	
3. Goldman and Gregory (2009)	USA	Two‐armed RCT; TAU control	Diagnosis of BPD; clinical settings—non‐specific	15	27.40	85.70% Caucasian; 90% female	**Dynamic deconstructive psychotherapy:** Individual^a^	Up to 52 sessions over 12 months^b^	5 therapists; 1 expert therapist, 4 third‐year trainee psychiatrists	Weekly group supervision. Biweekly individual supervision was used to review audio recordings to monitor treatment fidelity
4. Hirsh et al. (2012)	Canada	Two‐armed RCT; active control	People with a diagnosis of BPD and experience of suicidal behaviour and NSSI outpatient	**Total: 87** **DBT:** 43	**Total:** 31.41 **DBT:** 30.56	Not reported; 100% female	**DBT:** 60‐min individual sessions, 120‐min skills group and 120‐min phone coaching	Sessions delivered weekly over 1 year at two teaching hospitals^c^	25 therapists **DBT: 13 therapists** 3 psychiatrists, 4 PhD level psychologists, 5 master's level clinicians and 1 nurse	**DBT:** Weekly group supervision (2 h)
				**Active control:** 44	**Active control:** 32.25	Not reported	**Active control:** Individual general psychiatric management (includes dynamically informed psychotherapy)^a^		**Active control: 12 therapists** 8 psychiatrists, 1 PhD level psychologist, 1 master's level clinician and 2 nurses	**Active control:** Weekly group supervision (90 min)
5. Bryan et al. (2012)	USA	Cohort/longitudinal	Military Primary care clinic	497	37.14	54.10% Caucasian; 57.7% female	**CBT:** 30‐min individual sessions	Up to 8 sessions at a primary care clinic	22 therapists; 8 clinical psychologists (6 trainers and 2 externship trainees), 9 predoctoral clinical psychology interns and 5 social worker interns	Interns were trained under the supervision of clinical psychologists to deliver CBT. No further details on supervision reported
6. Perry et al. (2013)	Canada	Cohort/longitudinal	People with diagnoses of anxiety, depression and/or PD outpatient—psychiatry	53	30.90	Not reported; 77% female	**Long‐term dynamic psychotherapy:** Individual^a^	Up to 339 sessions over a median of 4.19 years at an outpatient clinic	22 therapists; psychiatrists, psychologists, social workers and advanced practice nurses; 20 were also psychoanalysts	No supervision groups or specific therapy manual used
7. Tsai et al. (2014)	Canada	Cohort/longitudinal	People with a diagnosis of depression who were outpatient/in the community	80	47.82	76.10% Caucasian; 73% female	**CBT for depression:** 120‐min group sessions	Up to 10 sessions over 10 weeks at an outpatient community mental health service/hospital	2 therapists; 1 clinical psychologist and 1 psychiatrist	Not reported
8. Bedics et al. (2015)	USA	Two‐armed RCT; active control	People with a diagnosis of BPD and experience of suicidal behaviour and NSSI in the community	**Total:** 101 **DBT:** 52	**Total:** 29.30	**Total:** 86.50% Caucasian; 100% female	**DBT:** 60‐min individual sessions and 150 min of group skills and telephone consultations per week	Sessions delivered over 1 year at university outpatient clinic and community practice^c^	37 therapists **DBT:** 15 (12 of whom had a doctoral degree)	**DBT:** Weekly group supervision
				**Active control:** 49			**Active control:** Community treatment by experts (eclectic/psychodynamic therapy)^a^		**Active control:** 25 (14 of whom had a doctoral degree)	**Active control:** Not required to attend supervision
9. Gysin‐Maillart et al. (2016)^d^	Switzerland	Two‐armed RCT; TAU control	People who had recently attempted suicide who are attending a psychiatry outpatient department	60	36.50	Not reported; 60% female	**Attempted Suicide Short Intervention Program (ASSIP):**Up to 90‐min individual sessions	3 sessions (4 if necessary) delivered weekly at an outpatient department	4 therapists; 1 psychiatrist and 3 clinical psychologists (2 of whom were experienced in clinical suicide prevention)	Regular supervision to review therapy video recordings to ensure therapy fidelity
10. Gysin‐Maillart et al. (2017)^d^	Switzerland	RCT; TAU control	People who had recently attempted suicide who are attending a psychiatry outpatient department	60	36.50	Not reported; 60% female	**ASSIP:** Up to 90‐min individual sessions	3 sessions (4 if necessary) delivered weekly at an outpatient department	4 therapists; 1 psychiatrist and 3 clinical psychologists (2 of whom were experienced in clinical suicide prevention)	Regular supervision to review therapy video recordings to ensure therapy fidelity
11. Plöderl et al. (2017)	Austria	Cohort/longitudinal	People who had attempted suicide and/or had suicidal ideation and were admitted to an inpatient ward	633	39.19	Not reported; 51% female	**Individual and group psychotherapeutic crisis intervention (eclectic, pan‐theoretical and flexible)** ^a^	Up to 15 weekly sessions over 3 weeks on the inpatient ward and up to 5 further follow‐up sessions over 6 months delivered at a clinic or via telephone	7 therapists; psychiatrists, psychotherapists/psychologists	Not reported
12. Rufino and Ellis (2018)	USA	Cohort/Longitudinal	People with diagnoses related to mood, anxiety and/or PD and suicidal thoughts and admitted to an inpatient ward	434	33.44	91.00% Caucasian; 53.5% female	**Individual therapy; psycho‐educational and therapeutic groups;** f**amily therapy** ^a^	Sessions delivered on an inpatient ward^c^	Not reported	Not reported
13. Ibrahim et al. (2018)^d^	USA	Two‐armed RCT; active control	People who were experiencing depression and suicidal thoughts recruited from a mix of clinical and non‐clinical settings	**Total:** 115 **Attachment‐based family therapy:** 60	**Total:** 14.96	**Total:** 28.70% Caucasian; 82.9% female	**Attachment‐based family therapy:** 90‐min individual and family therapy sessions	16 weekly sessions over 16 weeks delivered at a university research lab/intervention clinic	17 therapists; all at least master's level	Not reported
				**Active Control:** 55			**Active control:** Family‐enhanced non‐directive supportive therapy Individual^a^ sessions and 4 60‐min parent psycho‐educational sessions			
14. Haddock et al. (2019)	UK	Two‐armed RCT; TAU control	People with experiencing of suicidal thoughts and/or behaviours and admitted to an inpatient ward	24	33.88	91.67% Caucasian; 58% female	**Cognitive behavioural suicide prevention therapy:** Up to 70‐min individual sessions	20 sessions delivered over 6 months on an inpatient ward and followed up in the community	2 therapists; both clinical psychologists who met the British Association for Behavioural and Cognitive Psychotherapies minimum standards for CBT accreditation	Weekly supervision
15. Johnson et al. (2019)	USA	Two‐armed RCT; active control	Veterans who had recently attempted suicide and recently discharged from an inpatient ward	**Total:** 134 **Suicide‐focused assessment group therapy:** 69	**Total:** Not reported **Suicide Focused Assessment Group Therapy:** 47.72	70.90% Caucasian; 11.9% female	**Suicide‐focused assessment group therapy:** Group sessions^a^	**Suicide‐focused assessment group therapy:** Up to 12 weekly sessions delivered in an outpatient setting	2 therapists facilitated both group therapies; 1 clinical psychologist and 1 social worker	Observation and spot checks by the principal investigator ensured adherence and fidelity to suicide‐focused assessment group therapy
				**Active control:** 65	**Active control:** 48.33		**Active control:** Usual assessment group therapy Group sessions^a^	**Active control:** Up to 12 weekly sessions delivered in an outpatient setting		
16. Ryberg et al. (2019)	Norway	Two‐armed RCT; active control	People with ongoing suicidal ideation, intent, and behaviour in both inpatient and outpatient settings	**Total:** 78 **Collaborative Assessment and Management of Suicidality (CAMS):** 37	**Total:** 39.90 **CAMS:** 38.40	Not reported; 53% female	**CAMS with psychodynamic, cognitive or eclectic psychotherapy:** Up to 60‐min individual sessions	**CAMS:** A mean of 17.80 therapy sessions were attended weekly, of which 7.90 were CAMS specific. Therapy sessions were delivered in inpatient and outpatient settings. Number of sessions not predetermined	43 therapists; **CAMS:** 8 psychologists and 1 psychiatrist	**CAMS:** Once therapists were adherent to the CAMS procedure, supervision was available by request
				**Active control:** 41	**Active control:** 33.70		**Active control:** Psychodynamic, cognitive or eclectic psychotherapy up to 45‐min individual sessions	**Active control:** A mean of 14.6 weekly sessions delivered in inpatient and outpatient settings. Number of sessions not predetermined	**Active control:** 15 psychologists, 4 residents, 6 psychiatrists and 9 psychiatric nurses	**Active control:** Not reported
17. Stratton et al. (2020)	Canada	Two‐armed RCT; waitlist control	People with diagnosis of BPD; suicidal behaviour and NSSI in an outpatient setting	43	27.29	Not reported; 83.3% female	**DBT skills:** 120‐min group	20 weekly sessions delivered at a teaching hospital	5 therapists; 2 PhD, 3 MSW	Weekly group supervision
18. Huggett et al. (2021)	UK	Two‐armed RCT; TAU control	People with non‐affective psychosis‐related diagnoses; suicidal ideation and/or behaviour in both inpatient and outpatient settings	64	36.83	88% Caucasian; 43.75% female	**Cognitive behavioural suicide prevention therapy:** Up to 180‐min individual sessions	Up to 24 sessions delivered over 6 months in outpatient and inpatient settings	8 individuals who were clinical psychologists, mental health nurses and a social worker and met the British Association of Behavioural and Cognitive Psychotherapies minimum standards for CBT accreditation	Weekly group supervision, monthly individual supervision, and regular peer supervision to ensure and monitor therapy fidelity
19. Ibrahim et al. (2021)d	USA	Two‐armed RCT; active control	People who were experiencing depression and suicidal thoughts recruited from a mix of clinical and non‐clinical settings	118	14.96	28.7% Caucasian; 81.7% female	**Attachment‐based family therapy:** 90‐min individual and family therapy sessions	Up to 16 weekly sessions over 16 weeks delivered at a university research lab/intervention clinic	17 therapists; all at least master's level	Weekly supervision, which included live supervision and review of therapy tapes
							**Active control:** Family‐enhanced nondirective supportive therapy Individual^a^ sessions and 4 60‐min parent psychoeducation sessions			

^a^ Length of sessions not reported.

^b^ Setting not reported.

^c^ Number of sessions not reported.

^d^ Used the same RCT sample.

### 3.3 Measures of the therapeutic alliance and suicidal experiences

It is worth considering first ways in which the therapeutic alliance was measured and second ways in which suicidal experiences were measured and documented across studies (see Table 2).

**TABLE 2:** Details of the therapeutic alliance, suicidal ideation and suicide attempt measures used in included studies

	Therapeutic alliance measure			Suicidal experiences measures	
Study number and reference	Client rated	Therapist rated	Observer rated	Suicidal ideation measure	Suicide attempt measure
1. Shearin and Linehan (1992)	Structural Analysis of Social Behaviour INTREX form (Benjamin, 1988) Rated session by session, weekly, Sessions 1–31 (early–mid therapy)	Structural Analysis of Social Behaviour INTREX form (Benjamin, 1988) Rated session by session, weekly, Sessions 1–31 (early–mid therapy)	N/A	Measured using a daily diary card	Measured using a daily diary card
2. Turner (2000)	Helping Relationship Questionnaire (Haq; Luborsky, 1984) Rated at 6 months (mid‐therapy)	N/A	N/A	Beck Suicidal Ideation Scale (Beck et al., 1988) Measured at baseline, 6 and 12 months (pre‐, mid‐ and end of therapy)	Target behaviour ratings—frequency of parasuicide

3. Goldman and Gregory (2009)	N/A	N/A	Working Alliance Inventory Observer Short form (WAI‐O‐S; Tichenor & Hill, 1989; Tracey & Kokotovic, 1989) Rated at baseline, 3, 6 9 and 12 months (early, mid and end of therapy)	N/A	The Lifetime Parasuicide Count (Linehan & Comtois, 1996) Measured at baseline, 3, 6, 9 and 12 months (pre‐, mid‐ and end of therapy)
4. Hirsh et al. (2012)	Working Alliance Inventory (WAI; Horvath & Greenberg, 1989) Rated at baseline, 4, 8 and 12 months (early, mid and end of therapy)	N/A	N/A	N/A	Suicide Attempt Self‐Injury Interview (Linehan et al., 2006) Measured at baseline, 4, 8 and 12 months (pre‐, mid‐ and end of therapy)

5. Bryan et al. (2012)	The Therapeutic Bond Scale (CelestHealth Solutions, 2008) Rated after session 1 (early in therapy)	N/A	N/A	1 item from the Behavioral Health Measure‐20 (Kopta & Lowry, 2002) Measured session‐by‐session	N/A
6. Perry et al. (2013)	The Psychosocial Treatment Interview (PTI; Steketee et al., 1997) Measured every 6 months (early, mid and end of therapy)	N/A	Therapeutic Alliance Analogue Scales (Brysk, 1987) Rated 3 sessions around 1 months and 6 months (early in therapy)	Longitudinal Interval Follow‐up Evaluation (Keller et al., 1987) Adapted for the Study of Personality (Perry, 1990) measured at baseline and every 6–12 months (pre‐, mid‐ and end of therapy)	N/A
7. Tsai et al. (2014)	WAI (Horvath & Greenberg, 1986; Horvath & Greenberg, 1989) Rated after Sessions 1 and 5 (early and mid‐therapy)	N/A	N/A	Number of participants with recurring or current ideation at baseline	Number of participants who had previously attempted suicide at baseline
8. Bedics et al. (2015)	California Psychotherapy Alliance Scale (Gaston, 1991) Rated after Session 1 and at 4, 8 and 12 months (early, mid and end of therapy)	California Psychotherapy Alliance Scale (Gaston, 1991) Rated after Session 1 and at 4, 8 and 12 months (early, mid and end of therapy)	N/A	N/A	Suicide Attempt Self‐Injury Interview (Linehan et al., 2006) Measured at baseline, 4, 8 and 12 months (pre‐, mid‐ and end of therapy)

9. Gysin‐Maillart et al. (2016)^a^	Penn Haq–German version (Bassler et al., 1995; Luborsky, 1984) Rated after Sessions 1 and 3 (early and end of therapy)	N/A	N/A	Beck Scale for Suicidal Ideation (BSS) German version (Beck & Steer, 1991; Fidy, 2008) Measured at baseline, 6, 12, 18 and 24 months (pre‐therapy and follow‐up time points)	Demographic question and hospital records
10. Gysin‐Maillart et al. (2017)^a^	Penn Haq–German version (Bassler et al., 1995; Luborsky, 1984) Rated after Sessions 1 and 3 (early and end of therapy)	N/A	N/A	BSS German version (Beck & Steer, 1991; Fidy, 2008) Measured at baseline, 6 and 12 months (pre‐therapy and follow‐up time points)	BSS German version (Beck & Steer, 1991; Fidy, 2008) Measured at baseline, 6 and 12 months (pre‐therapy and follow‐up time points)
11. Plöderl et al. (2017)	WAI–Short Revised German Translation (Wilmers et al., 2008) Rated at intake and discharge from the inpatient ward (early and towards the end of therapy)	N/A	N/A	BSS (Beck & Steer, 1991) Measured at intake and discharge from the inpatient ward (early and towards the end of therapy)	BSS (Beck & Steer, 1991) Measured at intake and discharge from the inpatient ward (early and towards the end of therapy)
12. Rufino and Ellis (2018)	WAI (Horvath & Greenberg, 1989) Rated at admission, every 2 weeks and prior to discharge (early, mid and end of therapy)	N/A	N/A	Columbia Suicide Severity Rating Scale (Posner et al., 2011) Suicide Cognitions Scale (Bryan et al., 2014; Ellis & Rufino, 2015) Measured at admission, every 2 weeks and prior to discharge (early, mid and end of therapy)	Frequency of prior suicide attempts measured at admission to the inpatient ward (early in therapy)
13. Ibrahim et al. (2018)^a^	Therapeutic Alliance Quality Scale (Riemer et al., 2012) Rated session by session on a weekly basis, between Sessions 1 and 16 (early, mid and end of therapy)	N/A	N/A	Suicidal Ideation Questionnaire–Junior weekly (Reynolds & Mazza, 1999) Measured at baseline (pre‐therapy)	Suicide attempt history measured at baseline (pre‐therapy)

14. Haddock et al. (2019)	WAI (Horvath & Greenberg, 1989) Rated at Session 4 and end of therapy (early and end of therapy)	WAI (Horvath & Greenberg, 1989) Rated at Session 4 and end of therapy (early and end of therapy)	N/A	BSS (Beck et al., 1979) and Suicide Probability Scale (Cull & Gill, 1982) Measured at baseline, 6 week and 6 months (pre‐therapy, early therapy, and end of therapy)	Frequency of suicide attempts collected by a review of clinical records between randomization and 6 months (start to end of therapy)
15. Johnson et al. (2019)	WAI‐S (Hatcher & Gillaspy, 2006) Rated at 1 and 3 months (early and end of therapy)	N/A	N/A	BSS (Beck et al., 1979) Measured at baseline, 1 month and 3 months (pre‐therapy, early therapy, and end of therapy)	Suicide Attempt and Self‐Injury Count (Linehan & Comtois, 1999) Measured at baseline, 1 month and 3 months (pre‐therapy, early therapy and end of therapy)
16. Ryberg et al. (2019)	WAI‐S (Hatcher & Gillaspy, 2006) Rated after 3 weeks of therapy (early in therapy)	N/A	N/A	BSS (Beck et al., 1997) Measured at baseline, 6 and 12 months (pre‐therapy and follow‐up time points)	N/A
17. Stratton et al. (2020)	Group Session Rating Scale (GSRS; Duncan & Miller, 2007) Rated at baseline, 5, 10, 15 and 20 weeks and 3 months post‐intervention (pre‐therapy early, mid and end of therapy and follow up)	N/A	N/A	N/A	Lifetime Suicide Attempt and Self‐Injury Interview (Linehan & Comtois, 1996) Measured at baseline, 5, 10, 15 and 20 weeks and 3 months post‐intervention (pre‐therapy early, mid and end of therapy and follow‐up)
18. Huggett et al. (2021)	WAI‐SR (Hatcher & Gillaspy, 2006) Rated at Session 4 (early in therapy)	WAI‐SR (Hatcher et al., 2020) Measured at Session 4 (early in therapy)	N/A	Adult Suicidal Ideation Questionnaire (ASIQ; Reynolds, 1991) Measured at baseline and end of therapy (pre‐therapy and end of therapy)	Self‐reported frequency of Suicide Attempts over the previous 6 months measured at baseline and end of therapy (pre‐therapy and end of therapy)
19. Ibrahim et al. (2021)^a^	Therapeutic Alliance Quality Scale (Riemer et al., 2012) Rated at Session 4 (early in therapy)	N/A	N/A	Suicidal Ideation Questionnaire–Junior weekly (Reynolds & Mazza, 1999) Measured at 16 weeks (end of therapy)	N/A

^a^ Used the same RCT sample.

First, the most frequently used measure of therapeutic alliance was the Working Alliance Inventory (WAI; Horvath & Greenberg, 1989). The WAI was used in 10 studies, two of which measured both client and therapist perspectives; seven of which sampled client perspectives only; and one of which sampled independent observer ratings of client–therapist alliance. The remaining nine studies captured the client perspective of the therapeutic alliance by using seven different measures other than the WAI. Further, two studies used two different measures other than the WAI to capture the therapist perspectives of the alliance. The final study used an independent observer rated measure to assess the client and therapist alliance (Perry et al., 2013). It is important to consider who collects the therapeutic alliance measures from clients. This is because clients may not want to be seen as being critical of the therapist, which could impact on the quality of intervention delivery and therapeutic outcome (Lingiardi et al., 2016). Of the 19 studies in the current review, 17 measured client perspectives of the therapeutic alliance, with five out of those 17 being administered by independent researchers. Furthermore, two studies used independent observer ratings of therapy session video or audio recordings. Studies ranged from measuring the therapeutic alliance at one time point, that is, Session 1 (Bryan et al., 2012) or after 3 weeks (Ryberg et al., 2019) or 6 months (Turner, 2000), through to measurements taken across 16–31 session‐by‐session ratings using two therapeutic alliance measures (Ibrahim et al., 2018; Shearin & Linehan, 1992).

Second, there was considerable variability in measures of suicidal experiences. The Beck Scale for Suicide Ideation (Beck et al., 1988) was the most commonly used validated self‐report measure of suicidal experiences, whereas several different validated interview‐based measures and non‐validated measures were also used. Although some measures have the capacity to measure both suicidal ideation and self‐harm in addition to suicide attempts, it should be noted that most studies treated such variables as separate during the analysis in relation to the therapeutic alliance. However, the Suicide Probability Scale (SPS; Cull & Gill, 1982), which assessed a combination of suicidal ideation, negative thoughts, hopelessness and hostility, was used and analysed as a composite measure in one study (Haddock et al., 2019). Two studies included a population of adolescents and so used the Suicidal Ideation Questionnaire–Junior to examine suicidal ideation (Ibrahim et al., 2018, 2021).

Third, suicidal experiences were measured at several time points, including prior to taking part in psychotherapy (e.g. measured at baseline or admission to a mental health inpatient ward), during psychotherapy (e.g. measured session‐by‐session or early and mid‐therapy), towards the end or upon cessation of psychotherapy and at follow‐up time points.

### 3.4 The relationship between the therapeutic alliance in psychotherapy and suicidal experiences

This review focuses upon understanding the extent to which (1) suicidal experiences occurring pre‐therapy influenced the therapeutic alliance, (2) suicidal experiences are correlated with/related to the therapeutic alliance at the same time point during psychotherapy and (3) the therapeutic alliance developed during therapy affected suicidal experiences post‐therapy or at therapy cessation.

### 3.5 Suicidal experiences pre‐therapy as a predictor of the therapeutic alliance

A summary of analyses used and statistics produced by studies which examined suicidal experiences pre‐therapy as a predictor of the therapeutic alliance is presented in Table 3.

**TABLE 3:** Details of analyses used and statistics produced in included studies which examined suicidal experiences pre‐therapy as a predictor of the therapeutic alliance

	Suicidal experiences pre‐therapy as a predictor of the therapeutic alliance			
Study number and reference	Suicidal ideation pre‐therapy as a predictor of the therapeutic alliance	Suicide attempts pre‐therapy as a predictor of the therapeutic alliance	Change in suicidal ideation and behaviour combined as a predictor of change in the therapeutic alliance	Suicide attempts as a predictor of change in the therapeutic alliance over time
1. Shearin and Linehan (1992)	N/A	N/A	**Time series** Client χ^2^(8) = 29.46, *p* < .001 Therapist χ^2^(8) = 25.68, *p* < .001	N/A
3. Goldman and Gregory (2009)	N/A	**Spearman's correlation** Client *r* = −.04, *p* = .925	N/A	N/A
7. Tsai et al. (2014)	**Independent samples *t‐*test** Client Session 1: *t* = −.422, *df* = 59, *p* = .674 Session 5: *t* = −1.23, *df* = 50, *p* = .225	**Independent samples *t‐*test** Client Session 1: *t = *.439, *df =* 58, *p = *.662 Session 5: *t = *.388, *df =* 49, *p = *.700	N/A	N/A
10. Gysin‐Maillart et al. (2017)	N/A	**Bivariate correlations** Client *Session 1*: *r* = −.34, *p* = .008 Session 3: *r* = −.13, *p* = .340	N/A	N/A
11. Plöderl et al. (2017)	N/A	**Wilcoxon test** Client *Previous suicide attempt*: *M = 46.70* *No previous suicide attempt*: *M* = 48.59 *W* = 54,697, *N* = 633, *p* = .02	N/A	N/A
13. Ibrahim et al. (2018)	**Multiple hierarchical linear regression** Client β = −.04, *p* = .07, *SE* = .03, *df* = 100, *t* = −1.38	N/A	N/A	N/A
14. Haddock et al. (2019)	**Pearson's correlation** Client ideation: *r* = −.222, *n* = 17, *p* = .195 Potential: *r* = −.226, *n* = 17, *p* = .192 Therapist ideation: *r = .162*, *n = 22*, *p = .235* Potential: *r = .360*, *n = 22*, *p = .050*	N/A	N/A	N/A
15. Johnson et al. (2019)	**Path analysis** Client *IRR* = .73	N/A	N/A	N/A
17. Stratton et al. (2020)	N/A	**Bivariate correlation** *r* = −.10	N/A	**Logistic regression** *r* = −.17
18. Huggett et al. (2021)	**Pearson's correlation** Client *r*(57) = −.115, *p* = .386, 99% CI [−.43, 0.23] Therapist *r*(58) = −.034, *p* = .794, 99% CI [−.36, 0.30]^a^	**Independent samples *t*‐test** Client *t*(56) = −2.46, *p* = .023, 99% CI [−10.69, 0.36] Therapist *t*(57) = −1.34, *p* = .186, 99% CI [−5.51, 2.21]^a^	N/A	N/A

^a^ The authors amended the alpha level to .01 to minimize the probability of a Type 1 error occurring and to correct for multiple testing.

### 3.6 Suicidal ideation pre‐therapy as a predictor of the therapeutic alliance

Four studies consistently found that experience of suicidal ideation prior to psychotherapy was not significantly related to (Haddock et al., 2019; Huggett et al., 2021) and did not significantly predict (Ibrahim et al., 2018; Johnson et al., 2019) *client perceptions* of the therapeutic alliance at Session 4 or 1 month into therapy. A fifth study found that for people with and without suicidal ideation prior to therapy, there were no significant differences between alliance scores at Session 1 and Session 5 of psychotherapy (Tsai et al., 2014). A non‐significant relationship was also observed between a measure of suicide potential pre‐therapy and client therapeutic alliance at Session 4 (Haddock et al., 2019). Thus, the current evidence indicated that suicidal ideation prior to therapy did not significantly influence client perceptions of the therapeutic alliance early on in therapy.

Similarly, in two studies, suicidal ideation prior to therapy was not significantly related to *therapist perceptions* of the therapeutic alliance at Session 4 (Haddock et al., 2019; Huggett et al., 2021). Conversely, there was a moderate significant positive relationship between self‐reported suicide potential prior to therapy and the therapist view of the therapeutic alliance at Session 4, even though the sample size was small (Haddock et al., 2019). Hence, the evidence from this study suggests that clients with greater self‐reported suicide potential, which involved experiences of suicidal thoughts, hopelessness, negative self‐evaluations and hostility, were perceived by therapists as forming a *stronger* therapeutic alliance early on in therapy. This is despite a measure of suicidal ideation prior to therapy not relating to therapist views of the therapeutic alliance across two studies.

### 3.7 Suicide attempts pre‐therapy as a predictor of the therapeutic alliance

Six studies examined the extent to which lifetime suicide attempts or suicide attempts in the previous 6 months influenced the formation and maintenance of the therapeutic alliance from the perspective of the client or an observer. One study suggested that client perceptions of the therapeutic alliance at Session 1, which was held on admission to a mental health inpatient ward, were significantly lower in people who had previously attempted suicide compared to those who had not attempted suicide (Plöderl et al., 2017). In a second study, there was a moderate negative significant relationship between the number of suicide attempts prior to psychotherapy and therapeutic alliance measured at the first psychotherapy session (Gysin‐Maillart et al., 2017), but by the third session, this negative relationship had diminished. Notably, only three or four sessions were offered as part of this specific psychotherapy.

However, in two studies, there was a non‐significant relationship between number of suicide attempts prior to group psychotherapy and therapeutic alliance measured in Session 1 (Stratton et al., 2020; Tsai et al., 2014) and Session 5 (Tsai et al., 2014). In a third study, frequency of lifetime suicide attempts at baseline had no relationship with the therapeutic alliance after 3 months of psychotherapy (Goldman & Gregory, 2009). Furthermore, a fourth study found no evidence to suggest a significant difference in client nor therapist perceptions of the therapeutic alliance when clients had previously attempted suicide or not (Huggett et al., 2021).

In summary, clients who have attempted suicide prior to commencing psychotherapy have varied perceptions of the robustness of the therapeutic alliance at the first session but are still able to form a good therapeutic alliance with a psychotherapist at the outset of therapy.

### 3.8 Change in suicidal ideation and behaviour combined as a predictor of change in the therapeutic alliance

One study analysed suicidal ideation and behaviour as a composite variable (Shearin & Linehan, 1992). A time series approach was taken to analysing the session‐by‐session data over 7 months of psychotherapy. Experiences of the composite measure of suicide during therapy were significantly associated with client perceptions that the therapists were understanding and warm in the following week's therapy session (Shearin & Linehan, 1992).

### 3.9 Suicide attempts as a predictor of change in the therapeutic alliance over time

One study implicitly examined lifetime frequency of suicide attempts prior to group psychotherapy and whether this was related to change in the client perception of the therapeutic alliance over time (Stratton et al., 2020). Lifetime frequency of suicide attempts did not significantly correlate with change in therapeutic alliance over the course of group psychotherapy (Stratton et al., 2020).

### 3.10 Suicidal experiences as a correlate of the therapeutic alliance at the same time point during psychotherapy

A summary of analyses used and statistics produced by studies which examined suicidal experiences as a correlate of the therapeutic alliance at the same time point during psychotherapy is presented in Table 4.

**TABLE 4:** Details of analyses used and statistics produced in included studies which examined suicidal experiences as a correlate of the therapeutic alliance at the same time point during psychotherapy

	Suicidal experiences as a correlate of the therapeutic alliance at the same time point during psychotherapy	
Study number and reference	Suicidal ideation in relation to the therapeutic alliance at the same time point during therapy	Suicide attempts in relation to the therapeutic alliance at the same time‐point during therapy
3. Goldman and Gregory (2009)	N/A	**Spearman's correlation** Client *r* = .08, *p* = .851
6. Perry et al. (2013)	**Wilcoxon test** Client 1 month: *Z* = 1.83, *p* = .07; *Z* = 1.70, *p* = .09	N/A
11. Plöderl et al. (2017)	**Spearman's correlation** Client Session 1: *r* = −.19, *N* = 633, *p* < .01 Final session: *r* = −.36, *N* = 633, *p* = .01	N/A

### 3.11 Suicidal ideation in relation to the therapeutic alliance at the same time point during therapy

In the present review, experience of suicidal ideation measured during therapy was cross‐sectionally examined in relation to the therapeutic alliance during psychotherapy by only two studies. From the client's perception of the therapeutic alliance, one study found a small, negative relationship between suicidal ideation and therapeutic alliance at Session 1, which took place on admission to a crisis intervention and suicide prevention inpatient ward (Plöderl et al., 2017). A second study (Perry et al., 2013) found only trends towards a significant difference between the therapeutic alliance ratings, 1 month into psychotherapy again in people with and without suicidal ideation at this time point. Similarly, in the same study, therapist views of the therapeutic alliance (1 and 6 months into psychotherapy) and client views of the therapeutic alliance (6 months into psychotherapy) did not differ dependent on whether the client had or had not experienced suicidal ideation (Perry et al., 2013). Thus, the majority of the evidence indicates that experience of suicidal ideation during psychotherapy did not influence client or therapist perceptions of the therapeutic alliance early on or part way through psychotherapy.

A moderate negative relationship was observed between client perception of the therapeutic alliance and suicidal ideation towards the end of psychotherapy, that is, discharge from the mental health inpatient ward (Plöderl et al., 2017). Such finding suggests that clients who perceived the therapeutic alliance as stronger towards the end of psychotherapy experienced less severe suicidal thoughts.

In summary, the current literature suggests client and therapist perceptions of the therapeutic alliance early on, or part way through psychotherapy, are not related to client experiences of suicidal thoughts. Although, most notably in an inpatient population, towards the end of psychotherapy and final session on inpatient wards, clients who perceived the therapeutic alliance as stronger experienced less severe suicidal ideation.

### 3.12 Suicide attempts in relation to the therapeutic alliance at the same time point during therapy

In the present review, only one study (Goldman & Gregory, 2009) examined the cross‐sectional relationship between suicide attempts and the therapeutic alliance. The average of the observer‐rated therapeutic alliance had no significant relationship with the total frequency of suicide attempts, both collected over four time points during psychotherapy (Goldman & Gregory, 2009).

### 3.13 Therapeutic alliance as a predictor of prospective suicidal experiences during and post‐therapy

A summary of analyses used and statistics produced by studies which examined the therapeutic alliance as a predictor of prospective suicidal experiences during and post‐therapy is presented in Table 5.

**TABLE 5:** Details of analyses used and statistics produced in included studies which examined the therapeutic alliance as a predictor of prospective suicidal experiences during and post‐therapy

	Therapeutic alliance as a predictor of prospective suicidal experiences during and post‐therapy			
Study number and reference	Therapeutic alliance in relation to suicidal ideation post‐therapy	Therapeutic alliance as a predictor of prospective suicidal behaviour (e.g. suicide attempts and self‐harm) during and post‐therapy	Therapeutic alliance during psychotherapy in relation to predicting prospective changes in suicidal ideation over time	Therapeutic alliance during psychotherapy in relation to predicting change in suicidal behaviour (e.g. suicide attempts) over time
1. Shearin and Linehan (1992)	N/A	N/A	N/A	**Time series** Client χ^2^(8) = 25.68, *p* < .001 Therapist χ^2^(8) = 17.26, *p* < .05
2. Turner (2000)	**Canonical correlation** Alliance: Canonical coefficient = .628 Intervention: Canonical coefficient = .631 Therapy cessation suicidal ideation: Canonical coefficient = .84	**Canonical correlation** Therapy cessation suicide attempts and self‐harm (composite measure) Canonical coefficient = .80	N/A	N/A
3. Goldman and Gregory (2009)	N/A	**Predictive correlation** *r* = .36, *p* = .552	N/A	N/A
4. Hirsh et al. (2012)	N/A	N/A	N/A	**Multilevel modelling** Client *b* = −.01, *SE* = .01, t/chi‐square = 2.92 Reduction in suicide attempts *b* = −.05, *SE* = .02, *t* = 10.09, *p* < .05
5. Bryan et al. (2012)	N/A	N/A	**Repeated measures mixed linear Regression** Client *B* = .045, *SE* = .117, *p* = .702	N/A
6. Perry et al. (2013)	N/A	N/A	**Simple linear regression** Interactions *r*_s_ = −.45, *n* = 28, *p* = .02 Client *r*_s_ = −.18, *n* = 28, *p* = .38 Therapist *r*_s_ = −.24, *n* = 28, *p* = .24	N/A
8. Bedics et al. (2015)	N/A	N/A	N/A	**Hierarchical linear modelling** **Client** Changes in alliance *b* = −.12, *SE* = .10, *z* = −1.14, *p* = .26 Working capacity Suicide‐focused therapy *b* = −.35, *SE* = .16, *z* = −2.39, *p* < .02 Therapy without focus on suicide prevention *b* = .02, *SE* = .13, *z* = .17, *p* = .87 **Therapist** Overall alliance across both therapies *b* = −.31, *SE* = .10, *z* = −3.13, *p* < .005 Suicide‐focused therapy Overall alliance *b* = −.34, *SE* = .14, *z* = −2.38, *p* < .02 Client commitment *b* = −.28, *SE* = .11, *z* = −2.56, *p* < .02 Client working capacity *b* = −.26, *SE* = .12, *z* = −2.26, *p* < .03 Therapy without focus on suicide prevention Understanding and involvement *b* = −.43, *SE* = .14, *z* = −3.00, *p* < .003 Overall alliance *b* = −.27, *SE* = .14, *z* = −1.93, *p* = .05
9. Gysin‐Maillart et al. (2016) ¶	**Linear regression** Client 12‐month follow‐up: *t*57 = −3.02, *p* = .004; coefficient: −.26, R^2^ = .18 24‐month follow‐up: *t*57 = −3.11, *p* = .003; coefficient: −.21, R^2^ = .30	N/A	N/A	N/A
10. Gysin‐Maillart et al. (2017) ¶	**Stepwise multiple linear regression** Client β = −.334, R^2^ = .386, *p* = .004	N/A	N/A	N/A
11. Plöderl et al. (2017)	N/A	N/A	**Spearman's correlation (change score calculated as difference pre and post)** Client *r* = .05, *p* = .23	N/A
15. Johnson et al. (2019)	N/A	N/A	**Structural equation modelling** Client *IRR* = 1.04, *p* = .001	N/A
16. Ryberg et al. (2019)	N/A	N/A	**Mixed effects linear regression** Overall alliance 6‐month follow‐up: β = .38, *N* = 78, *p* = .039 Client–therapist bond 6‐month follow‐up β = .1.47, *N* = 78, *p* = .003 12‐month follow‐up β = 1.10, *N* = 78, *p* = .029	N/A
18. Huggett et al. (2021)	**Pearson's correlation** Client *r*(58) = −.22, *p* = .087, 99% CI [−.51, .11] Therapist *r*(58) = −.22, *p* = .087, 99% CI [−.51, .11] **Multiple hierarchical linear regression** Client Model 1: β = −.33, *t*(56) = −2.66, *p* = .010, 95% CI [−2.64, −.37] R^2^ = .110, *p* = .010 for Step 1 Model 2: β = −.28, *t*(55) = −2.51, *p* = .015, 95% CI [−2.29, −.26] R^2^ = .231, *p* = .001 for Step 1; ΔR^2^ = .078, *p* = .015 for Step 2 Model 3: β = −.27, *t*(53) = −2.34, *p* = .023, 95% CI [−2.23, −.18] R^2^ = .231, *p* = .001 for Step 1; ∆R^2^ = .037, *p* = .261 for Step 2; ∆R^2^ = .068, *p* = .023 for Step 3. WAI‐SR **Moderated linear regression** Client Interaction effect: *b* = .003, *t*(54) = 1.85, *p* = .07 Total number of minutes spent in therapy Short: *b* = −2.07, 95% CI [−3.40, −.74], *t* = −3.12, *p* = .003 Mean: *b* = − 1.14, 95% CI [−2.18, −.11], *t* = −2.20, *p* = .032 Long: = − .21, 95% CI [−1.76, 1.34], *t* = −.59, *p* = .560	**Independent samples *t*‐test** Client t*(*55) = −.72, *p* = .463, 99% CI [−9.62, 6.64] Therapist *t(*56) = .63, *p* = .529, 99% CI [−4.68, 6.36]	N/A	N/A
19. Ibrahim et al. (2021)	**Hierarchical linear models** Interaction between therapy adherence and client alliance in relation to suicidal ideation *t* (329) = −2.72, *p* < .01 ∆R^2^ = .02, ∆F (3, 329) = 2.80, *p* = .04	N/A	N/A	N/A

### 3.14 Therapeutic alliance in relation to suicidal ideation post‐therapy

Six studies examined the therapeutic alliance as perceived by the client in relation to suicidal ideation towards the end of psychotherapy or upon psychotherapy cessation and at follow‐up time points.

First, there were significant negative relationships between the therapeutic alliance early on in therapy and suicidal ideation, across three studies, at therapy cessation (Huggett et al., 2021), 6‐month follow‐up (Gysin‐Maillart et al., 2017), 12‐month follow‐up (Gysin‐Maillart et al., 2016; Gysin‐Maillart et al., 2017) and 24‐month follow‐up (Gysin‐Maillart et al., 2016). These findings remained when baseline confounding variables were controlled for, that is, suicidal ideation, depression and hopelessness (Huggett et al., 2021) and depression and the number of previous suicide attempts (Gysin‐Maillart et al., 2017).

A fourth study analysed the simultaneous impact of the therapeutic alliance and intervention upon suicidal ideation. Both the therapeutic alliance measured mid‐way (6 months) through therapy and difference between the intervention groups, that is, DBT and client‐centred therapy had a similar relationship with lower severity of suicidal ideation upon therapy cessation (Turner, 2000).

A fifth study investigated the therapeutic alliance as a moderator between therapy adherence and suicidal ideation upon therapy cessation. The interaction between good therapy adherence and the client perception a stronger therapeutic alliance was significantly correlated with lower frequency of suicidal thoughts (Ibrahim et al., 2021).

In contrast, a sixth study indicated that client perception of the therapeutic alliance measured early on in therapy was not significantly correlated to suicidal ideation upon therapy cessation when discharged from the inpatient ward and at 2 weeks and 6 months' post‐discharge (Rufino & Ellis, 2018). Furthermore, there was no evidence for a significant relationship between therapist views of the alliance and suicidal ideation upon therapy cessation (Huggett et al., 2021).

Overall, there is evidence to suggest that a more robust therapeutic alliance perceived by the client early on or mid‐way through a suicide‐focused psychotherapy may be related to less severe or less frequent suicidal ideation both at the end of therapy and at follow‐up time points, although this finding was not supported by all included studies.

### 3.15 Therapeutic alliance as a predictor of prospective suicidal behaviour (e.g. suicide attempts and self‐harm) during and post‐therapy

Three studies examined the relationship between the therapeutic alliance in psychotherapy and suicidal behaviour post‐therapy (Goldman & Gregory, 2009; Turner, 2000). The first study (Goldman & Gregory, 2009) found that the observer‐rated therapeutic alliance at 3 months did not significantly relate to suicide attempts mid‐way (6 months) through psychotherapy. Additionally, the second study suggested there were no significant differences in client nor therapist perceptions of the therapeutic alliance when clients had previously attempted suicide or not (Huggett et al., 2021). In contrast, the third study (Turner, 2000) found that client perceptions of the therapeutic alliance as stronger, at the mid‐way point (6 months) during therapy, were as important as the type of therapy (DBT or client‐centred therapy) being delivered, in terms of explaining the impact on suicidal behaviour outcome post‐therapy (composite measure of suicide attempts and self‐harm).

In summary, studies examining client‐, therapist‐ and observer‐rated therapeutic alliance have contradictory findings as to whether the therapeutic alliance is related to subsequent suicide attempts.

### 3.16 Therapeutic alliance during psychotherapy in relation to predicting prospective changes in suicidal ideation over time

Five studies examined to what extent the therapeutic alliance in psychotherapy predicted changes in suicidal ideation over time (Bryan et al., 2012; Johnson et al., 2019; Perry et al., 2013; Plöderl et al., 2017; Ryberg et al., 2019). All five studies described the method used to calculate rate of change scores.

One study provided evidence that observer ratings of a strong therapeutic alliance at 6 months into therapy resulted in reduced suicidal ideation. An observer rating of one component of the therapeutic alliance, namely, interactions between the client and therapist (e.g. collaborative discussions and establishing a rapport), had a medium negative significant relationship with frequency of suicidal ideation over a median duration of 4.19 years (Perry et al., 2013). In other words, if client–therapist interactions were rated as strong by *observers*, there was a greater reduction in suicidal ideation over time. However, such a relationship was not reported for *client* or *therapist* perceptions of the therapeutic alliance overall, respectively.

A second study suggested that a strong therapeutic alliance early on in therapy moderated the relationship between type of psychotherapy and severity of suicidal ideation at follow‐up time points (Ryberg et al., 2019). More specifically, interactions between the overall therapeutic alliance and psychotherapy condition were significantly related to reductions in severity of suicidal ideation at 6‐month follow‐up. Similarly, an interaction between one component of the therapeutic alliance, the client–therapist bond and psychotherapy condition was significantly related to improvement in suicidal ideation at both 6‐month and 12‐month follow‐up.

In contrast, a third study found that a one unit increase in the strength of the client perception of the therapeutic alliance at 1 month was significantly related to a 4% *increase* in severity of suicidal ideation at the same time point (Johnson et al., 2019). However, changes in the therapeutic alliance from 1 month to therapy cessation at 3 months were not related to changes in suicidal ideation severity at the end of therapy.

A fourth study indicated that the client perception of the therapeutic alliance measured early on in psychotherapy did not significantly influence subsequent changes in suicidal ideation after two to eight sessions of psychotherapy (Bryan et al., 2012), although one may question if such a number of sessions is sufficient when working with suicidal clients. A fifth study also observed no such relationship between client view of the early therapeutic alliance in psychotherapy delivered on a mental health inpatient ward and changes in severity of suicidal ideation over the course of up to 15 sessions of psychotherapy (Plöderl et al., 2017).

To summarize, no firm conclusions can be made as to whether the therapeutic alliance in psychotherapy predicts change in suicidal ideation over time.

### 3.17 Therapeutic alliance during psychotherapy in relation to predicting change in suicidal behaviour (e.g. suicide attempts) over time

Three studies investigated whether the therapeutic alliance during psychotherapy predicted change in suicidal behaviour over time (Bedics et al., 2015; Hirsh et al., 2012; Shearin & Linehan, 1992). All three studies reported analyses which were used to examine change in suicidal attempts/behaviour over time.

One study examined the therapeutic alliance in two types of psychotherapy; more specifically, one was suicide focused, and one not exclusively focused on reducing suicidal thoughts and behaviours (Bedics et al., 2015). For all clients, regardless of psychotherapy received, changes in the therapeutic alliance did not significantly predict changes in frequency of suicide attempts. However, there appeared to be a trend towards an interaction, whereby for clients who received a suicide‐focused psychotherapy, there was a significant negative relationship between clients' perception of their working capacity and frequency of suicide attempts over the course of 12 months of therapy (Bedics et al., 2015). This indicates that as perceptions of working capacity increased, subsequent suicide attempts reduced. However, there was no such relationship for clients who received psychotherapy without a focus upon suicide prevention. Moreover, no other aspect of the therapeutic alliance was significantly related to suicide attempts, for example, client commitment, therapist understanding and involvement and agreement on working strategy (Bedics et al., 2015). Additionally, a second study (Hirsh et al., 2012) found that the client view of the therapeutic alliance was not significantly related to frequency of suicide attempts over 1 year of psychotherapy. This result occurred even though suicide attempts significantly reduced over the same time period (Hirsh et al., 2012).

When considering therapist perceptions of the therapeutic alliance, irrespective of whether or not therapy was suicide focused, overall perception of the therapeutic alliance and each component of the therapeutic alliance (client working capacity, client commitment, working strategy consensus and therapist understanding and involvement) had significant negative relationships with suicide attempts over 1 year of psychotherapy (Bedics et al., 2015). Furthermore, such relationships were further scrutinized for therapies with and without a specific focus on suicide prevention, respectively. For therapists who delivered a suicide‐focused therapy, it appeared that the overall therapeutic alliance, along with client commitment and client working capacity, had a significant negative relationship with suicide attempts over 1 year of psychotherapy (Bedics et al., 2015). Therapists' perception of their understanding and involvement was not related to frequency of client suicide attempts for therapists conducting suicide‐focused therapy. However, when therapists provided a therapy which was not specifically focused on suicide prevention, an increase in therapist perception of their understanding and involvement and overall perception of the alliance significantly predicted a reduction in suicide attempts over 1 year of psychotherapy (Bedics et al., 2015).

Similarly, a third study found that improvements in both client and therapist perceptions of the therapeutic alliance were associated with a significant reduction in suicidal behaviour over 7 months of psychotherapy (Shearin & Linehan, 1992). However, the definition of suicidal behaviour in this study (Shearin & Linehan, 1992) was not provided.

The current literature tentatively suggests that one component of the client perception of the therapeutic alliance (working capacity) and therapist perceptions of the overall therapeutic alliance predict a reduction in subsequent suicide attempts over the course of psychotherapy. Additionally, the results of one study demonstrate that different components of the therapist‐rated therapeutic alliance (i.e. therapist understanding and involvement, client commitment and client working capacity) were related to a reduction in suicide attempts when therapists used different therapeutic modalities.

### 3.18 Study quality

Across studies, four scored affirmatively for six or seven of the seven CASP criteria (Gysin‐Maillart et al., 2016, 2017; Hirsh et al., 2012; Huggett et al., 2021), whereas two only scored one or two, respectively (Rufino & Ellis, 2018; Shearin & Linehan, 1992; see Table 6). It was noticeable that those studies which met between six and seven out of seven criteria for study quality were most likely to be RCTs and had an outpatient population and used validated measures of alliance and suicidal experiences. Six studies adopted a cohort design, whereas RCTs were used to collect data for the other 13 studies. Inherently, cohort studies are not as robust as RCTs in minimizing bias (Levin, 2006, 2007). Most studies (*n* = 16) had acceptable outcome measure retention rates or accounted for attrition in the analysis to mitigate against attrition bias. Overall, the majority of studies appeared to be of good methodological quality, with 14 of the 19 studies meeting at least four out of seven criteria.

**TABLE 6:** Quality assessment for included studies

CASP question	1. Did the trial address a clearly focused issue?	2. Was the exposure accurately measured to minimize bias?	3. Was the outcome accurately measured to minimize bias?	4. Have authors identified all important confounding factors?	5. Was the follow‐up of subjects complete enough?	6. Can the results be applied to the local population?	7. Are the benefits worth the harms and costs?
Adaption	Does the study examine the relationship between the therapeutic alliance and suicidal experiences?	Does the study systematically train therapists and monitor therapist fidelity?	Were measures of therapeutic alliance and suicidal experiences reliable and valid?	Have authors identified and controlled for at least age and gender as confounding factors?	Were retention rates acceptable or did the authors account for attrition in the analysis?	Can the results be generalized to a similar population as the study population?	Is the psychotherapy safe, that is, were adverse and serious adverse events monitored and assessed?
1. Shearin and Linehan (1992)	Y	UC	UC	N	UC	N	UC
2. Turner (2000)	Y	Y	UC	UC	Y	UC	UC
3. Goldman and Gregory (2009)	Y	Y	UC	UC	Y	N	UC
4. Hirsh et al. (2012)	Y	Y	Y	Y	Y	Y	UC
5. Bryan et al. (2012)	Y	UC	Y	Y	UC	Y	UC
6. Perry et al. (2013)	Y	N	Y	Y	Y	UC	UC
7. Tsai et al. (2014)	Y	UC	Y	UC	Y	Y	UC
8. Bedics et al. (2015)	Y	Y	Y	UC	Y	Y	UC
9. Gysin‐Maillart et al. (2016)^a^	Y	Y	Y	Y	Y	Y	UC
10. Gysin‐Maillart et al. (2017)^a^	Y	Y	Y	Y	Y	Y	UC
11. Plöderl et al. (2017)	Y	UC	Y	UC	Y	UC	UC
12. Rufino and Ellis (2018)	Y	UC	Y	N	N	N	UC
13. Ibrahim et al. (2018)	Y	UC	Y	Y	Y	Y	UC
14. Haddock et al. (2019)	Y	Y	Y	UC	Y	UC	Y
15. Johnson et al. (2019)	Y	UC	Y	UC	Y	Y	UC
16. Ryberg et al. (2019)	Y	Y	Y	Y	Y	UC	UC
17. Stratton et al. (2020)	Y	Y	Y	Y	Y	UC	UC
18. Huggett et al. (2021)	Y	Y	Y	Y	Y	Y	Y
19. Ibrahim et al. (2021)	Y	Y	Y	UC	Y	Y	UC

^a^ Used the same RCT sample.

Two key study quality criteria to consider when examining the therapeutic alliance are therapist training and fidelity (including supervision) and the safe delivery of therapy. It is essential for therapists to be trained and supervised to develop and maintain a therapeutic alliance with clients who have suicidal experiences in accordance with the therapy manual (e.g. CT [Brown et al., 2011], CBT [Pratt et al., 2016], DBT [Rizvi, 2011] and psychodynamic therapy [Weinberg et al., 2011]). Further, the occurrence of ruptures and harmful interactions during therapy have been posited as risk factors for adverse reactions in psychological therapy (Parry et al., 2016). As such, monitoring and assessing adverse events, such as suicide attempts, is vital to the safe delivery of therapy.

A particular strength pertaining to the quality of the data in the current review is that measures of therapy fidelity, including use of a therapy manual and supervision, were robust across 11 studies, lending reassurance to the findings. Furthermore, this could have positively, and consistently, influenced therapists' interaction with clients who were suicidal and thus the alliance. Therefore, such rigorous procedures may increase the likelihood that psychotherapy delivery, including the development of a strong therapeutic alliance, could be reproduced by future studies.

Information was omitted in four studies about how therapist fidelity was assessed and maintained despite providing details of therapist training (Bryan et al., 2012; Ibrahim et al., 2018; Plöderl et al., 2017; Tsai et al., 2014). Similarly, Johnson et al. (2019) reported therapist training and adherence procedures for administering the suicide status form but did not report on training or adherence for the group therapy. One study reported that neither a specific therapy manual nor supervision groups were used due to the naturalistic study design (Perry et al., 2013). Consequently, there was ambiguity around whether there was consistency in therapists' approach to developing and maintaining the therapeutic alliance and the delivery of therapy in the context of suicidal experiences. A stronger approach would have been to ensure a psychotherapy manual was followed, therapist fidelity was assessed and maintained through regular supervision and a validated psychotherapy adherence scale, and such procedures were reported transparently.

The safety of the psychotherapy delivery, including the therapeutic alliance, in the context of working with people who have suicidal experiences was only monitored and assessed by two studies (Haddock et al., 2019; Huggett et al., 2021). This constitutes best practice in order to prevent possible harm in therapy. It remains unclear as to whether the other studies included in the review did monitor and assess adverse events and/or if it is an issue of insufficient reporting in the published papers.

Two studies lacked transparency and did not report psychometric properties for measures of therapeutic alliance and suicidal experiences (Goldman & Gregory, 2009; Turner, 2000). This could indicate selective reporting bias, which creates uncertainty as to whether measures of alliance and suicidal experiences were reliable and valid in these studies and undermines the credibility of the study findings. However, the majority of studies (*n* = 16) demonstrated that measures of the therapeutic alliance and suicidal experiences were valid and reliable, which contributes to the trustworthiness and possible generalizability of the review findings.

All studies had at least one skew in their samples. One study sample was skewed towards people with no previous suicide attempts at both Session 1 (85.00%) and Session 5 (84.31%; Tsai et al., 2014). Other study samples were skewed towards individuals who were under 40 years of age (*n* = 16), female (*n* = 10) and Caucasian (*n* = 8). Furthermore, possible reporting bias was identified across eight studies as no details regarding ethnicity were given. Thus, whilst findings may generalize to a similar population, it is unclear if study findings were representative across different ages, gender identities and ethnic groups.

Power was possibly compromised in four studies where the primary study aim did not include examining the relationship between the therapeutic alliance and suicidal experiences (Goldman & Gregory, 2009; Haddock et al., 2019; Ibrahim et al., 2018; Stratton et al., 2020). Two studies included variables of suicidal ideation (Ibrahim et al., 2018) or suicide attempts (Stratton et al., 2020) as one of nine covariates. Furthermore, the Goldman and Gregory (2009) study was considerably underpowered to detect a significant relationship with data only available for eight or five participants in respective analyses. Such concerns about power contribute to queries over the generalisability of the results for four studies. Moreover, and despite there being sufficient power to detect an effect size, the effect size was small, which may be qualified by the large sample size and may compromise clinical relevance (Plöderl et al., 2017).

The Shearin and Linehan (1992) study met only one of the quality assessment criteria and several factors were not addressed. Furthermore, only four participants were included in the study. Consequently, the study findings have limited generalizability and should be interpreted with caution.

A particular barrier to adequately assessing study quality was the lack of consistent reporting across several areas of potential bias. A number of studies did not describe therapist training and fidelity, psychometric properties for measures of therapeutic alliance and suicidal experiences and whether the psychotherapy was safely conducted. Furthermore, issues such as low retention rates for the measure of suicidal ideation, that is, 32% (Rufino & Ellis, 2018), short or unclear follow‐up timeframe (Bryan et al., 2012; Plöderl et al., 2017) and unclear therapy timeframe (Ryberg et al., 2019) were identified. Such reporting and retention, follow‐up and therapy timeframe problems across several studies may interfere with generalizability and transferability of review findings. Therefore, future studies could benefit from improving study quality in the aforementioned areas.

## 4 DISCUSSION

The aim of the present systematic review was to examine the nature of the relationship between the therapeutic alliance in psychotherapy and suicidal experiences by investigating suicidal ideation and attempts. This was achieved by examining the influence of suicidal experiences pre‐therapy upon the therapeutic alliance, the relationship between suicidal experiences and the therapeutic alliance when both measured at the same time point during psychotherapy and also by considering how the therapeutic alliance impacts upon suicidal experiences occurring post‐therapy. Overall, included studies were heterogeneous and provided varied evidence for the relationship between the therapeutic alliance and suicidal experiences as predictors, correlates and outcomes.

The current review suggests that some clients who experience suicidal ideation at the time of, and prior to, the initial psychotherapy session may experience barriers to forming and maintaining a therapeutic alliance, most noticeably when located on an inpatient ward (Plöderl et al., 2017). Furthermore, previous suicide attempts may influence the formation of the therapeutic alliance during the first session of a psychotherapy designed for people who had recently attempted suicide but may not hinder the development of a therapeutic alliance as psychotherapy progresses (Gysin‐Maillart et al., 2017). Possible explanations could be that clients may initially have concerns about building trust and how confidentiality is maintained during psychotherapy in the context of suicidal thoughts and acts (Awenat et al., 2018; Blanchard & Farber, 2020), both of which are integral to developing and maintaining an alliance with a therapist. Furthermore, more severe or frequent suicidal experiences have been found to be related to higher rates of self‐stigma in people experiencing a range of mental health problems (Latalova et al., 2014). Given such self‐stigmatizing beliefs, clients may be apprehensive about the potential emotional and practical consequences of disclosing suicidal experiences (Awenat et al., 2018; Blanchard & Farber, 2020). This suggests that therapists need to take particular care in discussing confidentiality limits with clients. Furthermore, therapists should provide reassurance to clients that they need only discuss what they initially feel comfortable with disclosing (Pratt et al., 2016).

In contrast, experiences of suicidal ideation and previous suicide attempts did not seem to influence the formation of the therapeutic alliance early on in some psychotherapies. Comparably, qualitative findings suggest that directly addressing suicidal experiences may not detrimentally influence the development of the therapeutic alliance, but highlight that sensitive listening, responding at appropriate times and creating a safe space for therapeutic discussion are the key facilitators of therapeutic alliance formation as perceived by both clients and therapists (Østlie et al., 2018). Therefore, the present review findings are comparable to the wider therapeutic alliance literature which presents mixed findings on the impact of the severity of mental health problems and the strength of therapeutic alliance throughout the course of psychotherapy (Strunk et al., 2010; Zilcha‐Mano et al., 2014).

The therapeutic relationship has been identified by both adolescents and adults with suicidal experiences as an important aspect of psychotherapies (Awenat et al., 2017; Paulson & Everall, 2003; Winter et al., 2014). The present review highlights that a robust therapeutic alliance early on in psychotherapy may be related to less severe suicidal thoughts at 6, 12 and 24‐month follow‐up time points and a reduction in suicidal ideation at 6‐ and 12‐month follow‐up and over a median of 4.19 years. Furthermore, the alliance mid‐way through therapy may be related to fewer suicidal thoughts and fewer suicide attempts at therapy cessation (after 12 months). Additionally, improvements in alliance over the course of therapy may be related to a reduction in suicide attempts over 7–12 months (mid‐way through to end of therapy). These findings are consistent with the wider alliance–outcome literature, which has found that the strength of the therapeutic alliance is related to positive clinical outcomes upon therapy cessation (Flückiger et al., 2018). Conversely, this is not a consistent finding across all studies in the current review (Bryan et al., 2012; Goldman & Gregory, 2009; Hirsh et al., 2012; Plöderl et al., 2017; Rufino & Ellis, 2018). Such findings may be attributed to several methodological limitations. For instance, and perhaps most importantly, some studies had insufficient power to detect a significant relationship. Other studies used suboptimal cohort designs, and many were not transparent in assessment of therapist training, fidelity and supervision. This could introduce ambiguity as to how the alliance was developed and maintained and how it is used to facilitate change in therapy may vary between therapeutic approaches, for example, DBT (Rizvi, 2011) and psychodynamic therapy (Weinberg et al., 2011).

In terms of the therapist perception of the therapeutic alliance, only four studies measured this perspective (Bedics et al., 2015; Haddock et al., 2019; Huggett et al., 2021; Shearin & Linehan, 1992). One study also included an observational subscale related to indicators of therapist views of the therapeutic alliance (Perry et al., 2013). All five of these studies evaluated psychotherapies which focused on reducing suicidal experiences. Therapists felt able to form a better therapeutic alliance with those who had experienced more severe suicide potential prior to therapy. The therapist view of the overall therapeutic alliance was not only related to a subsequent reduction in suicide attempts, but some specific components of the therapeutic alliance appeared to be more strongly related to an amelioration in suicide attempts. For instance, in a suicide‐specific psychotherapy, greater emphasis was placed upon client commitment and service user working capacity, whereas in a psychotherapy not focused solely on suicidal experiences, therapist understanding, and involvement were highlighted (Bedics et al., 2015). The aforementioned components of the therapeutic alliance may be indicative of different foci across different therapeutic modalities and so may influence how the therapeutic alliance is perceived by therapists (e.g. DBT [Rizvi, 2011] and psychodynamic therapy [Weinberg et al., 2011]).

### 4.1 Strengths

There are three key strengths of the current review. First, the review presents a comprehensive appraisal of the literature examining the relationship between therapeutic alliance in psychotherapy and suicidal experiences. Second, it was inclusive of all individuals who have suicidal experiences and all individual and group psychotherapies, with no restrictions placed on therapeutic alliance and suicidal experience measures. Third, efforts were made to identify any potential papers missed in the systematic search by forward and backward citation chaining, which ensured a thorough search was conducted. Furthermore, the authors requested data analyses from peer‐reviewed studies, which may help to alleviate publication and outcome reporting biases (Sterne et al., 2017) and subsequent ‘file‐drawer’ issues (Rosenthal, 1979). This inclusivity ensured that as much of the available literature as possible was reviewed.

### 4.2 Limitations

Four limitations of the current systematic review should be taken into account when considering whether the findings apply to current practice across different healthcare systems. First, the variety of populations, psychotherapies and therapeutic alliance and suicidal experience measures contributed to difficulties in interpreting and synthesizing this literature. Additionally, there were a variety of sample sizes across studies, with just under one‐third of studies involving a small sample size (*n* < 50; Kim, 2013) and so possibly being underpowered to detect an effect. The search was also restricted to English language papers, and not all corresponding authors were able to provide analyses between therapeutic alliance and suicidal experiences, and grey literature, such as dissertations, were not included. This suggests that there may be literature missing from the review due to publication and outcome reporting biases along with the ‘file‐drawer’ issue (Rosenthal, 1979). Such limitations also pose an issue for generalizability and representation of possible data.

Second, people with non‐affective psychosis, bipolar disorder and eating disorders were under‐represented in the present review. The literature to date has not focused on the relationship between the therapeutic alliance in psychotherapy and experiences of people with bipolar diagnoses (Flückiger et al., 2018). However, a narrative review posited that a robust therapeutic alliance in psychotherapy perceived by people with non‐affective psychosis may be associated with a reduction in distressing symptoms of psychosis and increased self‐esteem (Shattock et al., 2018). Furthermore, a recent meta‐analysis of 20 studies found a reciprocal relationship between improvements in experiences of eating disorders and a more robust therapeutic alliance (Graves et al., 2017). Suicidal experiences are also prevalent in people with non‐affective psychosis (Taylor et al., 2010), bipolar disorder (Owen et al., 2018) and eating disorders (Smith et al., 2018). Therefore, little is known about the relationship between the therapeutic alliance in psychotherapy and suicidal experiences in these populations.

Third, an inherent limitation, highlighted by the quality appraisal, is the lack of reporting on the safety of psychotherapy delivery and the client–therapist alliance. Issues such as harmful client–therapist interactions and unresolved ruptures in the therapeutic alliance, along with therapists not recognizing and repairing therapeutic alliance ruptures, could be risk factors for adverse reactions to psychotherapy (Parry et al., 2016). This reinforces the notion of the therapeutic alliance as integral to therapeutic outcomes. As per the Good Clinical Practice guidelines (World Health Organization [WHO], 2002) and UK policy framework for health and social care research (Health Research Authority [HRA], 2017), a study‐specific procedure should be developed and implemented to identify, assess and report adverse and serious adverse events in relation to the therapeutic alliance in psychotherapy. Furthermore, the CONSORT (Consolidated Standards of Reporting Trials) statement outlines specific guidance on reporting adverse events in peer‐reviewed publications (Ioannidis et al., 2004; Moher et al., 2010). Only two studies in the current review followed WHO, HRA and CONSORT guidelines and provided a comprehensive account of recording adverse events and assessing relatedness to trial procedures and psychotherapy (Haddock et al., 2019; Huggett et al., 2021).

Fourth, there was limited reporting on therapist characteristics and perspectives in the present review. Research examining influence of therapist factors on the therapeutic alliance and how this in turn impacts on outcome is lacking. An additional omission from the literature is the congruence between therapist and client perspectives of the therapeutic alliance. A meta‐synthesis found that both clients and therapists perceived that facilitators of effective psychotherapy include therapists showing respect, understanding and being non‐judgemental (Winter et al., 2014). Such qualities are reflective of Rogers' (1957, 1965) seminal work and the person‐centred literature whereby the constructs of empathy (Elliott et al., 2018), unconditional positive regard (Farber et al., 2018) and genuineness (Kolden et al., 2018) have been linked to psychotherapy outcome. Furthermore, client–therapist agreement on the therapeutic alliance can be integral to positive (Marmarosh & Kivlighan, 2012) or negative therapeutic outcomes (Rubel et al., 2018), although dependent on which outcome measure is used to assess alliance (Igra et al., 2020).

### 4.3 Clinical implications

Although there are contradictory findings as to whether suicidal experiences prior to psychotherapy influenced the client perception of the therapeutic alliance, it is important for therapists to be mindful of the possibility that suicidal experiences prior to psychotherapy could act as a barrier for clients to building a therapeutic alliance within the first session (Gysin‐Maillart et al., 2017; Plöderl et al., 2017). Such difficulties in forming a therapeutic alliance may be due to client concern about building trust, maintenance of confidentiality, the power dynamic and imbalance of control and both the perceived emotional and practical consequences of discussing suicidal experiences (Awenat et al., 2018; Blanchard & Farber, 2020; Jobes & Ballard, 2011). Moreover, client perceptions and expectations of relationships may also influence the therapeutic alliance (Zilcha‐Mano, 2017).

A number of key aspects of client characteristics should also be considered, namely, age, gender identity, sexual orientation, ethnicity, employment status and education. These factors may influence motivation to engage in and complete psychotherapy and form meaningful therapeutic relationships (Behn et al., 2018; Chang & Yoon, 2011; Meier et al., 2005; Sharf et al., 2010; Wintersteen et al., 2005). Therefore, it is imperative that therapists are not only trained in engaging clients and building the therapeutic alliance but also attune to client perceptions of relationships and concerns about discussing suicidal experiences during therapy.

Since there are cultural differences in both the perception of suicidal experiences (Colucci & Too, 2014) and psychotherapy (Edge & Lemetyinen, 2019), therapists should undergo necessary training to increase cultural competence by learning about and reflecting on cultural and ethnic issues. Most notably, the focus should be on alleviating the potential impact differences in ethnicity, sexual orientation, gender identity and socio‐economic status between the therapist and client may have on building a therapeutic alliance and therapeutic change (Behn et al., 2018; Cardemil & Battle, 2003; Chang & Yoon, 2011; Vasquez, 2007).

There may also be inherent power imbalances in a client–therapist relationship which could influence the therapeutic alliance. Such power imbalances may be amplified when working with clients with suicidal experiences due to expectations imposed by society which suggest that it is the therapist's responsibility to keep clients safe (Jobes & Ballard, 2011). Through both training and supervision, it is recommended that therapists take particular care in discussing confidentiality limits with clients, along with placing emphasis on engagement and fostering trust throughout therapy (Pratt et al., 2016; Rizvi, 2011; Tarrier et al., 2013). More specifically, therapists should work to dispel myths about potential disclosures of suicidal experiences, along with addressing and/or openly considering any difficulties clients may foresee in relation to developing a therapeutic relationship in the context of suicide, in order to reassure clients and promote a safe environment to discuss suicidal experiences. Furthermore, training and supervision should be used to ensure therapists are aware of potential power imbalance and attempt to create an egalitarian power dynamic by taking an empathetic and collaborative approach, where clients are encouraged to share their story (Elliott & Greenberg, 2007; Jobes & Ballard, 2011; Pratt et al., 2016).

### 4.4 Future directions

Considering the findings and limitations of the current review, there are five important recommendations for future research studies. First, there is an opportunity to develop existing studies, including RCTs, which examine the feasibility or effectiveness of psychotherapies and ensure that the therapeutic alliance is both measured and examined in relation to suicidal experiences. It is recommended that the relationship between therapeutic alliance and suicidal experiences is reported as part of the main outcome paper of psychotherapy trials. Second, all future psychotherapy research studies adhere to WHO, HRA and CONSORT guidelines to improve transparency in monitoring and reporting adverse events to ensure the safety of participants with suicidal experiences in psychotherapy research studies. Third, given the lack of representation of people with non‐affective psychosis, bipolar disorder and eating disorders in the current review and to address such a gap in the literature, future studies should investigate the therapeutic alliance in psychotherapy in relation to suicidal experiences in people with non‐affective psychosis, bipolar disorder and eating disorders. Fourth, it appears there is a remarkable omission from the literature whereby studies have not examined the influence of therapist characteristics (i.e. age, gender identity, sexual orientation, ethnicity, professional background and length of experience) and client characteristics (i.e. age, gender identity, sexual orientation, ethnicity, employment status and education) on the formation and maintenance of the therapeutic alliance in the context of discussing suicidal thoughts and acts in psychotherapy. Therefore, future research should investigate whether therapist and client characteristics interact with the relationship between therapeutic alliance and suicidal experiences prior to therapy, during therapy and after therapy. It is also recommended that studies consistently measure and examine both client and therapist perception of the therapeutic alliance.

## 5 CONCLUSIONS

In summary, the current review provides an overview of the relationship between the therapeutic alliance and suicidal experiences as correlates, predictors and outcomes across a range of therapeutic modalities. The results highlight that it remains unclear how much impact suicidal experiences prior to and during psychotherapy may have upon the formation and maintenance of the therapeutic alliance. However, there is stronger evidence to suggest the therapeutic alliance during psychotherapy may be related to a reduction in future suicidal experiences. The present review highlighted several gaps and inconsistencies in the literature and made several recommendations for clinical practice and future research.

To conclude, few psychotherapy and suicidal experience studies have measured the therapeutic alliance, and even fewer have examined the relationship between the therapeutic alliance in psychotherapy and suicidal experiences. Future psychotherapy studies should more consistently examine the relationship between the therapeutic alliance and suicidal experiences prior to, during and after psychotherapy.

## CONFLICT OF INTERESTS

DP and PG were part of a team who developed a treatment manual which has been published as a book (Tarrier et al., 2013). This published work is under copyright. PG, GH, DP and CH are funded to work on the CARMS (Cognitive AppRoaches to coMbatting Suicidality) trial by the Efficacy and Mechanism Evaluation (EME) Programme, an MRC and NIHR partnership (Gooding et al., 2020). The CARMS trial is testing the efficacy of a Cognitive Behavioural Suicide Prevention (CBSP) Therapy in people experiencing non‐affective psychosis and suicidal thoughts and behaviours. At the time the current study was conducted, CH was the trial manager. PG and GH are co‐principal investigators and DP is a co‐investigator on the CARMS trial. CH, PG, GH and DP published a paper examining the relationship between the therapeutic alliance in CBSP and suicidal experiences (pre‐ and post‐therapy; Huggett et al., 2021). The Huggett et al. (2021) study was conducted alongside the present review, addressed some limitations from the existing literature and has been included in the current review. PG, GH, DP and CH previously worked on the INSITE trial, which was funded by the NIHR RfPB programme (Haddock et al., 2019). INSITE tested the feasibility and acceptability of CBSP for inpatients with suicidal experiences. This trial is an included study. CH has previously worked for a mental health charity in the North West of England and has published a research article with this affiliation. JQ has declared that she has no conflicts of interest.

## Data Availability

Data sharing not applicable to this article as no datasets were generated or analysed during the current study.

## APPENDIX A.

Summary of headings from the data extraction table

Study title, authors, year, journal, country of study, study design, inclusion and exclusion criteria, sample size in therapy arm, study setting, intervention details, therapeutic alliance measure and when this was collected, suicidal experience (ideation, plans and urges) measure and when this was collected, suicidal experience (attempts) measure and when this was collected, suicide death(s), other relevant measures, sample demographics (age, gender, sexual orientation, ethnicity, presenting mental health problem(s), education status, employment status, other relevant sample demographics), therapist characteristics (number of therapists, age, gender, profession/qualification(s), length of experience, other relevant characteristics), therapy delivery characteristics (maximum number of sessions to be attended, number of sessions attended, cancelled and not attended, length of sessions, setting of therapy, other relevant delivery characteristics), and analysis (summary of influence of predictor, quantitative analysis used, descriptive findings, correlation/regression findings—service user ratings, correlation/regression findings—therapist ratings, correlation/regression findings—observer ratings and any other relevant findings).

## References

[cpp2726-bib-0001] Awenat, Y. F. , Peters, S. , Gooding, P. A. , Pratt, D. , Shaw‐Núñez, E. , Harris, K. , & Haddock, G. (2018). A qualitative analysis of suicidal psychiatric inpatients views and expectations of psychological therapy to counter suicidal thoughts, acts and deaths. BMC Psychiatry, 18(1). 10.1186/s12888-018-1921-6 PMC6192165 30326878

[cpp2726-bib-0002] Awenat, Y. F. , Shaw‐Núñez, E. , Kelly, J. , Law, H. , Ahmed, S. , Welford, M. , Tarrier, N. , & Gooding, P. A. (2017). A qualitative analysis of the experiences of people with psychosis of a novel cognitive behavioral therapy targeting suicidality. Psychosis, 9(1), 38–47. 10.1080/17522439.2016.1198827

[cpp2726-bib-0112] Bassler, M. , Potratz, B. , & Krauthauser, H. (1995). Der “Helping Alliance Questionnaire” (HAQ) von Luborsky. Möglichkeiten zur Evaluation des therapeutischen Prozesses von stationärer Psychotherapie. Psychotherapeut, 40(1), 23–32.

[cpp2726-bib-0120] Beck, A. T. , & Steer, R. A. (1991). Manual for Beck scale for suicide ideation. In Psychological Corporation. Psychological Corporation.

[cpp2726-bib-0122] Beck, A. T. , Brown, G. K. , & Steer, R. A. (1997). Psychometric characteristics of the scale for suicide with psychiatric outpatients. Behaviour Research and Therapy, 35(11), 1039–1046. 10.1016/S0005-7967(97)00073-9 9431735

[cpp2726-bib-0109] Beck, A. T. , Kovacs, M. , & Weissman, A. (1979). Assessment of suicidal intention: The Scale for Suicide Ideation. Journal of Consulting and Clinical Psychology, 47(2), 343–352. 10.1037/0022-006X.47.2.343 469082

[cpp2726-bib-0003] Beck, A. T. , Steer, R. A. , & Ranieri, W. F. (1988). Scale for suicide ideation: Psychometric properties of a self‐report version. Journal of Clinical Psychology, 44(4), 499–505. 10.1002/1097-4679(198807)44:4<499::AID-JCLP2270440404>3.0.CO;2-6 3170753

[cpp2726-bib-0004] Bedics, J. D. , Atkins, D. C. , Harned, M. S. , & Linehan, M. M. (2015). The therapeutic alliance as a predictor of outcome in dialectical behavior therapy versus nonbehavioral psychotherapy by experts for borderline personality disorder. Psychotherapy, 52(1), 67–77. 10.1037/a0038457 25751116

[cpp2726-bib-0005] Behn, A. , Davanzo, A. , & Errázuriz, P. (2018). Client and therapist match on gender, age, and income: Does match within the therapeutic dyad predict early growth in the therapeutic alliance? Journal of Clinical Psychology, 74(9), 1403–1421. 10.1002/jclp.22616 29573351

[cpp2726-bib-0132] Benjamin, L. S. (1988). SASB short form user's manual. INTREX Interpersonal Institute, Inc.

[cpp2726-bib-0006] Bertelsen, M. , Jeppesen, P. , Petersen, L. , Thorup, A. , Øhlenschlæger, J. , Le Quach, P. , Christensen, T. Ø. , Krarup, G. , J⊘rgensen, P. , & Nordentoft, M. (2007). Suicidal behaviour and mortality in first‐episode psychosis: The OPUS trial. British Journal of Psychiatry, 191(SUPPL. 51), s140–s146. 10.1192/bjp.191.51.s140 18055932

[cpp2726-bib-0007] Beutler, L. E. (2009). Making science matter in clinical practice: Redefining psychotherapy. Clinical Psychology: Science and Practice, 16(3), 301–317. 10.1111/j.1468-2850.2009.01168.x

[cpp2726-bib-0008] Blanchard, M. , & Farber, B. A. (2020). “It is never okay to talk about suicide”: Patients' reasons for concealing suicidal ideation in psychotherapy. Psychotherapy Research, 30(1), 124–136. 10.1080/10503307.2018.1543977 30409079

[cpp2726-bib-0009] Booth, A. , Harris, J. , Croot, E. , Springett, J. , Campbell, F. , & Wilkins, E. (2013). Towards a methodology for cluster searching to provide conceptual and contextual “richness” for systematic reviews of complex interventions: Case study (CLUSTER). BMC Medical Research Methodology, 13(1), 118. 10.1186/1471-2288-13-118 24073615 PMC3819734

[cpp2726-bib-0010] Brown, G. K. , Wenzel, A. , & Rudd, M. D. (2011). Cognitive therapy for suicidal patients. In K. Michel & D. A. Jobes (Eds.), Building a therapeutic alliance with the suicidal patient (pp. 273–291). American Psychological Association.

[cpp2726-bib-0011] Bryan, C. J. , Corso, K. A. , Corso, M. L. , Kanzler, K. E. , Ray‐Sannerud, B. , & Morrow, C. E. (2012). Therapeutic alliance and change in suicidal ideation during treatment in integrated primary care settings. Archives of Suicide Research, 16(4), 316–323. 10.1080/13811118.2013.722055 23137221

[cpp2726-bib-0133] Bryan, C. J. , Rudd, D. M. , Wertenberger, E. , Etienne, N. , Ray‐Sannerud, B. N. , Morrow, C. E. , Peterson, A. L. , & Young‐Mccaughon, S. (2014). Improving the detection and prediction of suicidal behavior among military personnel by measuring suicidal beliefs: An evaluation of the Suicide Cognitions Scale. Journal of Affective Disorders, 159, 15–22. 10.1016/j.jad.2014.02.021 24679384

[cpp2726-bib-0129] Brysk, B. (1987). Treatment alliance in a character‐disordered and affective‐disordered population: Scale development, correlates and outcome two to three years after intake. Rutgers, the State University of New Jersey.

[cpp2726-bib-0012] Calati, R. , & Courtet, P. (2016). Is psychotherapy effective for reducing suicide attempt and non‐suicidal self‐injury rates? Meta‐analysis and meta‐regression of literature data. Journal of Psychiatric Research, 79, 8–20. 10.1016/j.jpsychires.2016.04.003 27128172

[cpp2726-bib-0013] Cardemil, E. V. , & Battle, C. L. (2003). Guess who's coming to therapy? Getting comfortable with conversations about race and ethnicity in psychotherapy. Professional Psychology: Research and Practice, 34(3), 278–286. 10.1037/0735-7028.34.3.278

[cpp2726-bib-0137] CelestHealth Solutions. (2008). Clinical report manual. CelestHealth Solutions.

[cpp2726-bib-0014] Centers for Disease Control and Prevention . (2020). Underlying cause of death 1999–2018 on CDC WONDER online database. Multiple Cause of Death Files, 1999–2018, National Center for Health Statistics. https://wonder.cdc.gov/controller/datarequest/D76;jsessionid=A55B91CE45AFB8D8C6A21D16BA0D252F. Accessed August 16, 2020.

[cpp2726-bib-0015] Chang, D. F. , & Yoon, P. (2011). Ethnic minority clients' perceptions of the significance of race in cross‐racial therapy relationships. Psychotherapy Research, 21(5), 567–582. 10.1080/10503307.2011.592549 21756191

[cpp2726-bib-0016] Chesney, E. , Goodwin, G. M. , & Fazel, S. (2014). Risks of all‐cause and suicide mortality in mental disorders: A meta‐review. World Psychiatry, 13(2), 153–160. 10.1002/wps.20128 24890068 PMC4102288

[cpp2726-bib-0017] Colucci, E. , & Too, L. S. (2014). Culture, cultural meanings, and suicide among people from migrant and refugee backgrounds. In D. D. van Bergen , A. H. Montesinos , & M. Schouler‐Ocak (Eds.), Suicidal behavior of immigrants and ethnic minorities in Europe (pp. 115–136). Hogrefe Publishing.

[cpp2726-bib-0018] Critical Appraisal Skills Programme . (2018). CASP cohort study checklist. https://casp-uk.net/casp-tools-checklists/. Accessed August 16, 2020.

[cpp2726-bib-0019] Crits‐Christoph, P. , Gibbons, M. B. C. , Hamilton, J. , Ring‐Kurtz, S. , & Gallop, R. (2011). The dependability of alliance assessments: The alliance‐outcome correlation is larger than you might think. Journal of Consulting and Clinical Psychology, 79(3), 267–278. 10.1037/a0023668 21639607 PMC3111943

[cpp2726-bib-0020] Cull, J. G. , & Gill, W. S. (1982). Suicide probability scale (SPS). https://www.wpspublish.com/sps-suicide-probability-scale

[cpp2726-bib-0021] DeRubeis, R. J. , & Feeley, M. (1990). Determinants of change in cognitive therapy for depression. Cognitive Therapy and Research, 14(5), 469–482. 10.1007/BF01172968

[cpp2726-bib-0130] Duncan, B. L. , & Miller, S. D. (2007). The group session rating scale. Author.

[cpp2726-bib-0022] Dunster‐Page, C. , Haddock, G. , Wainwright, L. , & Berry, K. (2017). The relationship between therapeutic alliance and patient's suicidal thoughts, self‐harming behaviors and suicide attempts: A systematic review. Journal of Affective Disorders, 223, 165–174. 10.1016/j.jad.2017.07.040 28755624

[cpp2726-bib-0023] Edge, D. , & Lemetyinen, H. (2019). Psychology across cultures: Challenges and opportunities. Psychology and Psychotherapy: Theory, Research and Practice, 92(2), 261–276. 10.1111/papt.12229 31001925

[cpp2726-bib-0024] Elliott, R. , Bohart, A. C. , Watson, J. C. , & Murphy, D. (2018). Therapist empathy and client outcome: An updated meta‐analysis. Psychotherapy, 55(4), 399–410. 10.1037/pst0000175 30335453

[cpp2726-bib-0025] Elliott, R. , & Greenberg, L. S. (2007). The essence of process‐experiential/emotion‐focused therapy. American Journal of Psychotherapy, 61(3), 241–254. 10.1176/appi.psychotherapy.2007.61.3.241 17985528

[cpp2726-bib-0118] Ellis, T. E. , & Rufino, K. A. (2015). A psychometric study of the suicide cognitions scale with psychiatric inpatients. Psychological Assessment, 27(1), 82–89. 10.1037/pas0000028 25285716

[cpp2726-bib-0026] Falkenström, F. , Granström, F. , & Holmqvist, R. (2013). Therapeutic alliance predicts symptomatic improvement session by session. Journal of Counseling Psychology, 60(3), 317–328. 10.1037/a0032258 23506511

[cpp2726-bib-0027] Farber, B. A. , Suzuki, J. Y. , & Lynch, D. A. (2018). Positive regard and psychotherapy outcome: A meta‐analytic review. Psychotherapy, 55(4), 411–423. 10.1037/pst0000171 30335454

[cpp2726-bib-0114] Fidy, R. (2008). Psychologische Suizidalitäts‐Diagnostik im Internet. Universität Zürich.

[cpp2726-bib-0028] Flückiger, C. , Del, A. C. , Wampold, B. E. , & Horvath, A. O. (2018). The alliance in adult psychotherapy: A meta‐analytic synthesis. Psychotherapy, 55(4), 316–340. 10.1037/pst0000172 29792475

[cpp2726-bib-0135] Gaston, L. (1991). Reliability and criterion‐related validity of the california psychotherapy alliance scales‐patient version. Psychological Assessment, 3(1), 68–74. 10.1037/1040-3590.3.1.68

[cpp2726-bib-0029] Gibbons, M. B. C. , Crits‐Christoph, P. , de la Cruz, C. , Barber, J. P. , Siqueland, L. , & Gladis, M. (2003). Pretreatment expectations, interpersonal functioning, and symptoms in the prediction of the therapeutic alliance across supportive‐expressive psychotherapy and cognitive therapy. Psychotherapy Research, 13(1), 59–76. 10.1093/ptr/kpg007 22475163

[cpp2726-bib-0030] Goldman, G. A. , & Gregory, R. J. (2009). Preliminary relationships between adherence and outcome in dynamic deconstructive psychotherapy. Psychotherapy, 46(4), 480–485. 10.1037/a0017947 22121844

[cpp2726-bib-0124] Gooding, P. A. , Pratt, D. , Awenat, Y. , Drake, R. , Elliott, R. , Emsley, R. , Huggett, C. , Jones, S. , Kapur, N. , Lobban, F. , Peters, S. , Haddock, G. (2020). A psychological intervention for suicide applied to non‐affective psychosis: the CARMS (Cognitive AppRoaches to coMbatting Suicidality) randomised controlled trial protocol. BMC Psychiatry, 20(1), 306. 10.1186/s12888-020-02697-8 32546129 PMC7298803

[cpp2726-bib-0031] Graves, T. A. , Tabri, N. , Thompson‐Brenner, H. , Franko, D. L. , Eddy, K. T. , Bourion‐Bedes, S. , Brown, A. , Constantino, M. J. , Flückiger, C. , Forsberg, S. , Isserlin, L. , Couturier, J. , Karlsson, G. P. , Mander, J. , Teufel, M. , Mitchell, J. E. , Crosby, R. D. , Prestano, C. , Satir, D. A. , … Thomas, J. J. (2017). A meta‐analysis of the relation between therapeutic alliance and treatment outcome in eating disorders. International Journal of Eating Disorders, 50(4), 323–340. 10.1002/eat.22672 28152196

[cpp2726-bib-0032] Gysin‐Maillart, A. , Schwab, S. , Soravia, L. , Megert, M. , & Michel, K. (2016). A novel brief therapy for patients who attempt suicide: A 24‐months follow‐up randomized controlled study of the attempted suicide short intervention program (ASSIP). PLoS Medicine, 13(3), e1001968. 26930055 10.1371/journal.pmed.1001968 PMC4773217

[cpp2726-bib-0033] Gysin‐Maillart, A. C. , Soravia, L. M. , Gemperli, A. , & Michel, K. (2017). Suicide ideation is related to therapeutic alliance in a brief therapy for attempted suicide. Archives of Suicide Research, 21(1), 113–126. 10.1080/13811118.2016.1162242 26984644

[cpp2726-bib-0034] Haddock, G. , Pratt, D. , Gooding, P. A. , Peters, S. , Emsley, R. , Evans, E. , Kelly, J. , Huggett, C. , Munro, A. , Harris, K. , Davies, L. , & Awenat, Y. (2019). Feasibility and acceptability of suicide prevention therapy on acute psychiatric wards: Randomized controlled trial. BJPsych Open, 5(1), e14. 10.1192/bjo.2018.85 30762509 PMC6381415

[cpp2726-bib-0116] Hatcher, R. L. , Lindqvist, K. , & Falkenström, F. (2020). Psychometric evaluation of the Working Alliance Inventory—Therapist version: Current and new short forms. Psychotherapy Research, 30(6), 706–717. 10.1080/10503307.2019.1677964 31621525

[cpp2726-bib-0119] Hatcher, R. L. , & Gillaspy, J. A. (2006). Development and validation of a revised short version of the Working Alliance Inventory. Psychotherapy Research, 16(1), 12–25. 10.1080/10503300500352500

[cpp2726-bib-0035] Health Research Authority . (2017). UK policy framework for health and social care research. https://www.hra.nhs.uk/planning-and-improving-research/policies-standards-legislation/uk-policy-framework-health-social-care-research/

[cpp2726-bib-0036] Higgins, J. P. T. , & Green, S. (2011). Cochrane handbook for systematic reviews of interventions (5.1.0). Cochrane Collaboration.

[cpp2726-bib-0037] Hirsh, J. B. , Quilty, L. C. , Bagby, R. M. , & McMain, S. F. (2012). The relationship between agreeableness and the development of the working alliance in patients with borderline personality disorder. Journal of Personality Disorders, 26(4), 616–627. 10.1521/pedi.2012.26.4.616 22867511

[cpp2726-bib-0038] Horvath, A. O. (2006). The alliance in context: Accomplishments, challenges, and future directions. Psychotherapy, 43(3), 258–263. 10.1037/0033-3204.43.3.258 22122094

[cpp2726-bib-0111] Horvath, A. O. , & Greenberg, L. S. (1986). The development of the Working Alliance Inventory. In L. S. Greenberg & W. M. Pinsof (Eds.), The psychotherapeutic process: A research handbook (pp. 529–556). Guilford Press.

[cpp2726-bib-0039] Horvath, A. O. , & Greenberg, L. S. (1989). Development and validation of the working alliance inventory. Journal of Counseling Psychology, 36(2), 223–233. 10.1037/0022-0167.36.2.223

[cpp2726-bib-0040] Huggett, C. , Gooding, P. A. , Haddock, G. , & Pratt, D. (2021). The relationship between the therapeutic alliance and suicidal experiences in people with psychosis receiving therapy. International Journal of Environmental Research and Public Health, 18(20), 10706. 10.3390/ijerph182010706 34682451 PMC8535896

[cpp2726-bib-0041] Ibrahim, M. , Jin, B. , Russon, J. , Diamond, G. , & Kobak, R. (2018). Predicting alliance for depressed and suicidal adolescents: The role of perceived attachment to mothers. Evidence‐Based Practice in Child and Adolescent Mental Health, 3(1), 42–56. 10.1080/23794925.2018.1423893

[cpp2726-bib-0042] Ibrahim, M. , Levy, S. , Gallop, B. , Ewing, S. K. , Hogue, A. , Chou, J. , Diamond, G. , Gallop, B. , Krauthamer Ewing, S. , Chou, J. , Hogue, A. , Chou, J. , & Diamond, G. (2021). Therapist adherence to two treatments for adolescent suicide risk: Association to outcomes and role of therapeutic Alliance. Family Process. 10.1111/famp.12660 33904589

[cpp2726-bib-0043] Igra, L. , Lavidor, M. , Atzil‐Slonim, D. , Arnon‐Ribenfeld, N. , de Jong, S. , & Hasson‐Ohayon, I. (2020). A meta‐analysis of client‐therapist perspectives on the therapeutic alliance: Examining the moderating role of type of measurement and diagnosis. European Psychiatry, 63(1). 10.1192/J.EURPSY.2020.67 PMC7372164 32594927

[cpp2726-bib-0044] Ioannidis, J. P. A. , Evans, S. J. W. , Gøtzsche, P. C. , O'Neill, R. T. , Altman, D. G. , Schulz, K. , & Moher, D. (2004). Better reporting of harms in randomized trials: An extension of the CONSORT statement. Annals of Internal Medicine, 141(10), 781–788. 10.7326/0003-4819-141-10-200411160-00009 15545678

[cpp2726-bib-0045] Jobes, D. A. , & Ballard, E. (2011). The therapist and the suicidal patient. In K. Michel & D. A. Jobes (Eds.), Building a therapeutic alliance with the suicidal patient (pp. 51–61). American Psychological Association.

[cpp2726-bib-0046] Johnson, J. , Gooding, P. , & Tarrier, N. (2008). Suicide risk in schizophrenia: Explanatory models and clinical implications, the schematic appraisal model of suicide (SAMS). Psychology and Psychotherapy: Theory, Research and Practice, 81, 55–77. 10.1348/147608307X244996 17919360

[cpp2726-bib-0047] Johnson, L. L. , O'Connor, S. S. , Kaminer, B. , Gutierrez, P. M. , Carney, E. , Groh, B. , & Jobes, D. A. (2019). Evaluation of structured assessment and mediating factors of suicide‐focused group therapy for veterans recently discharged from inpatient psychiatry. Archives of Suicide Research, 23(1), 15–33. 10.1080/13811118.2017.1402722 29220609

[cpp2726-bib-0048] Joiner, T. E. , & Silva, C. (2012). Why people die by suicide: Further development and tests of the interpersonal‐psychological theory of suicidal behavior. In P. R. Shaver & M. Mikulincer (Eds.), Meaning, mortality, and choice: The social psychology of existential concerns (pp. 325–336). American Psychological Association.

[cpp2726-bib-0049] Katrak, P. , Bialocerkowski, A. E. , Massy‐Westropp, N. , Kumar, V. S. S. , & Grimmer, K. A. (2004). A systematic review of the content of critical appraisal tools. BMC Medical Research Methodology, 4(1), 22. 10.1186/1471-2288-4-22 15369598 PMC521688

[cpp2726-bib-0131] Keller, M. B. , Lavori, P. W. , Friedman, B. , Nielsen, E. , Endicott, J. , Mcdonald Scott, P. , & Andreasen, N. C. (1987). The longitudinal interval follow‐up evaluation: A comprehensive method for assessing outcome in prospective longitudinal studies. Archives of General Psychiatry, 44(6), 540–548. 10.1001/archpsyc.1987.01800180050009 3579500

[cpp2726-bib-0050] Kim, H.‐Y. (2013). Statistical notes for clinical researchers: Assessing normal distribution (2) using skewness and kurtosis. Restorative Dentistry & Endodontics, 38(1), 52–54. 10.5395/rde.2013.38.1.52 23495371 PMC3591587

[cpp2726-bib-0051] Kolden, G. G. , Wang, C. C. , Austin, S. B. , Chang, Y. , & Klein, M. H. (2018). Congruence/genuineness: A meta‐analysis. Psychotherapy, 55(4), 424–433. 10.1037/pst0000162 30335455

[cpp2726-bib-0134] Kopta, S. M. , & Lowry, J. L. (2002). Psychometric evaluation of the behavioral health questionnaire‐20: A brief instrument for assessing global mental health and the three phases of psychotherapy outcome. Psychotherapy Research, 12(4), 413–426. 10.1093/ptr/12.4.413

[cpp2726-bib-0052] Latalova, K. , Prasko, J. , Kamaradova, D. , Ociskova, M. , Cinculova, A. , Grambal, A. , Kubinek, R. , Mainerova, B. , Smoldasova, J. , Tichackova, A. , & Sigmundova, Z. (2014). Self‐stigma and suicidality in patients with neurotic spectrum disorder‐A cross sectional study. Neuroendocrinology Letters, 35(6), 474–480. 25433850

[cpp2726-bib-0053] Levin, K. A. (2006). Study design IV: Cohort studies. Evidence‐Based Dentistry, 7(2), 51–52. 10.1038/sj.ebd.6400407 16858385

[cpp2726-bib-0054] Levin, K. A. (2007). Study design VII. Randomized controlled trials. Evidence‐Based Dentistry, 8(1), 22–23. 10.1038/sj.ebd.6400473 17380181

[cpp2726-bib-0055] Liberati, A. , Altman, D. G. , Tetzlaff, J. , Mulrow, C. , Gøtzsche, P. C. , Ioannidis, J. P. A. , Clarke, M. , Devereaux, P. J. , Kleijnen, J. , & Moher, D. (2009). The PRISMA statement for reporting systematic reviews and meta‐analyses of studies that evaluate healthcare interventions: Explanation and elaboration. BMJ (Clinical Research Ed.), 339. 10.1136/bmj.b2700 PMC2714672 19622552

[cpp2726-bib-0121] Linehan, M. M. , & Comtois, K. (1996). Lifetime parasuicide history. University of Washington, Seattle, WA, Unpublished work.

[cpp2726-bib-0108] Linehan, M. M. , & Comtois, K. A. (1999). Suicide attempt and self injury count. University of Washington.

[cpp2726-bib-0123] Linehan, M. M. , Comtois, K. A. , Brown, M. Z. , Heard, H. L. , & Wagner, A. (2006). Suicide Attempt Self‐Injury Interview (SASII): Development, reliability, and validity of a scale to assess suicide attempts and intentional self‐injury. Psychological Assessment, 18(3), 303–312. 10.1037/1040-3590.18.3.303 16953733

[cpp2726-bib-0056] Lingiardi, V. , Holmqvist, R. , & Safran, J. D. (2016). Relational turn and psychotherapy research. Contemporary Psychoanalysis, 52(2), 275–312. 10.1080/00107530.2015.1137177

[cpp2726-bib-0057] Luborsky, L. (1976). Helping alliance in psychotherapy. In J. L. Cleghhorn (Ed.), Successful psychotherapy (pp. 92–116). Brunner/Mazel.

[cpp2726-bib-0127] Luborsky, L. (1984). Principles of psychoanalytic psychotherapy: A manual for supportive‐expressive treatment. Basic Books.

[cpp2726-bib-0058] Marmarosh, C. L. , & Kivlighan, D. M. (2012). Relationships among client and counselor agreement about the working alliance, session evaluations, and change in client symptoms using response surface analysis. Journal of Counseling Psychology, 59(3), 352–367. 10.1037/a0028907 22774865

[cpp2726-bib-0059] Meier, P. S. , Donmall, M. C. , Barrowclough, C. , McElduff, P. , & Heller, R. F. (2005). Predicting the early therapeutic alliance in the treatment of drug misuse. Addiction, 100(4), 500–511. 10.1111/j.1360-0443.2005.01031.x 15784065

[cpp2726-bib-0060] Moher, D. , Hopewell, S. , Schulz, K. F. , Montori, V. , Gøtzsche, P. C. , Devereaux, P. J. , Elbourne, D. , Egger, M. , & Altman, D. G. (2010). CONSORT 2010 explanation and elaboration: Updated guidelines for reporting parallel group randomized trials. BMJ (Clinical Research Ed.), 340, 869. 10.1136/bmj.c869 PMC2844943 20332511

[cpp2726-bib-0061] Muran, J. C. , Safran, J. D. , Gorman, B. S. , Samstag, L. W. , Eubanks‐Carter, C. , & Winston, A. (2009). The relationship of early alliance ruptures and their resolution to process and outcome in three time‐limited psychotherapies for personality disorders. Psychotherapy, 46(2), 233–248. 10.1037/a0016085 22122620

[cpp2726-bib-0062] Nock, M. K. , & Kessler, R. C. (2006). Prevalence of and risk factors for suicide attempts versus suicide gestures: Analysis of the national comorbidity survey. Journal of Abnormal Psychology, 115(3), 616–623. 10.1037/0021-843X.115.3.616 16866602

[cpp2726-bib-0063] O'Connor, R. C. , & Kirtley, O. J. (2018). The integrated motivational‐volitional model of suicidal behaviour. Philosophical Transactions of the Royal Society B: Biological Sciences, 373, 20170268. 10.1098/rstb.2017.0268 PMC6053985 30012735

[cpp2726-bib-0064] Office for National Statistics . (2019). Suicides in the UK: 2018 registrations. https://www.ons.gov.uk/peoplepopulationandcommunity/birthsdeathsandmarriages/deaths/bulletins/suicidesintheunitedkingdom/2018registrations. Accessed August 16, 2020.

[cpp2726-bib-0065] Østlie, K. , Stänicke, E. , & Haavind, H. (2018). A listening perspective in psychotherapy with suicidal patients: Establishing convergence in therapists and patients private theories on suicidality and cure. Psychotherapy Research, 28(1), 150–163. 10.1080/10503307.2016.1174347 27238607

[cpp2726-bib-0066] Owen, R. , Dempsey, R. , Jones, S. , & Gooding, P. (2018). Defeat and entrapment in bipolar disorder: Exploring the relationship with suicidal ideation from a psychological theoretical perspective. Suicide and Life‐threatening Behavior, 48(1), 116–128. 10.1111/sltb.12343 28276599

[cpp2726-bib-0067] Parry, G. D. , Crawford, M. J. , & Duggan, C. (2016). Iatrogenic harm from psychological therapies‐time to move on. British Journal of Psychiatry, 208, 210–212. 10.1192/bjp.bp.115.163618 26932481

[cpp2726-bib-0068] Paulson, B. L. , & Everall, R. D. (2003). Suicidal adolescents: Helpful aspects of psychotherapy. Archives of Suicide Research, 7(4), 309–321. 10.1080/713848939

[cpp2726-bib-0125] Perry, J. C. (1990). Use of longitudinal data to validate personality disorders. In J. M. Oldham (Ed.), Personality disorders: New perspectives on diagnostic validity (pp. 25–40). American Psychiatric Press.

[cpp2726-bib-0069] Perry, J. C. , Bond, M. , & Presniak, M. D. (2013). Alliance, reactions to treatment, and counter‐transference in the process of recovery from suicidal phenomena in long‐term dynamic psychotherapy. Psychotherapy Research, 23(5), 592–605. 10.1080/10503307.2013.809560 23937543

[cpp2726-bib-0070] Plöderl, M. , Kunrath, S. , Cramer, R. J. , Wang, J. , Hauer, L. , & Fartacek, C. (2017). Sexual orientation differences in treatment expectation, alliance, and outcome among patients at risk for suicide in a public psychiatric hospital. BMC Psychiatry, 17. 10.1186/s12888-017-1337-8 PMC5433065 28506219

[cpp2726-bib-0128] Posner, K. , Brown, G. K. , Stanley, B. , Brent, D. A. , Yershova, K. V. , Oquendo, M. A. , Currier, G. W. , Melvin, G. A. , Greenhill, L. , Shen, S. , & Mann, J. J. (2011). The Columbia‐suicide severity rating scale: Initial validity and internal consistency findings from three multisite studies with adolescents and adults. American Journal of Psychiatry, 168(12), 1266–1277. 10.1176/appi.ajp.2011.10111704 22193671 PMC3893686

[cpp2726-bib-0071] Pratt, D. , Gooding, P. A. , Kelly, J. A. , Johnson, J. , & Tarrier, N. (2016). Case formulation in suicidal behaviour. In N. Tarrier & J. Johnson (Eds.), Case formulation in cognitive behaviour therapy: The treatment of challenging and complex cases (2nd ed.) (pp. 265–283). Routledge.

[cpp2726-bib-0072] Reynolds, C. , Simms, J. , Webb, K. , Corry, M. , McDermott, B. , Ryan, M. , Shannon, M. , & Dyer, K. F. W. (2017). Client factors that predict the therapeutic alliance in a chronic, complex trauma sample. Traumatology, 23(4), 294–302. 10.1037/trm0000114

[cpp2726-bib-0126] Reynolds, W. M. (1991). ASIQ, Adult Suicidal Ideation Questionnaire: Professional Manual. Psychological Assessment Resources.

[cpp2726-bib-0136] Reynolds, W. M. , & Mazza, J. J. (1999). Assessment of suicidal ideation in inner‐city children and young adolescents: Reliability and validity of the suicidal ideation questionnaire‐JR. School Psychology Review, 28(1), 17–30.

[cpp2726-bib-0115] Riemer, M. , Athay, M. M. , Bickman, L. , Breda, C. , Kelley, S. D. , & Vides De Andrade, A. R. (2012). The peabody treatment progress battery: History and methods for developing a comprehensive measurement battery for youth mental health. Administration and Policy in Mental Health and Mental Health Services Research, 39(1–2), 3–12. 10.1007/s10488-012-0404-1 22421933 PMC4229686

[cpp2726-bib-0073] Rizvi, S. L. (2011). The therapeutic relationship in dialectical behavior therapy for suicidal individuals. In K. Michel & D. A. Jobes (Eds.), Building a therapeutic alliance with the suicidal patient (pp. 255–271).

[cpp2726-bib-0074] Rogers, C. R. (1957). The necessary and sufficient conditions of therapeutic personality change. Journal of Consulting Psychology, 21(2), 95–103. 10.1037/h0045357 13416422

[cpp2726-bib-0075] Rogers, C. R. (1965). The therapeutic relationship: Recent theory and research. Australian Journal of Psychology, 17(2), 95–108. 10.1080/00049536508255531

[cpp2726-bib-0076] Rosella, L. , Bowman, C. , Pach, B. , Morgan, S. , Fitzpatrick, T. , & Goel, V. (2016). The development and validation of a meta‐tool for quality appraisal of public health evidence: Meta quality appraisal tool (MetaQAT). Public Health, 136, 57–65. 10.1016/j.puhe.2015.10.027 26993202

[cpp2726-bib-0077] Rosenthal, R. (1979). The file drawer problem and tolerance for null results. Psychological Bulletin, 86(3), 638–641. 10.1037/0033-2909.86.3.638

[cpp2726-bib-0078] Rubel, J. A. , Bar‐Kalifa, E. , Atzil‐Slonim, D. , Schmidt, S. , & Lutz, W. (2018). Congruence of therapeutic bond perceptions and its relation to treatment outcome: Within‐and between‐dyad effects. Journal of Consulting and Clinical Psychology, 86(4), 341–353. 10.1037/ccp0000280 29389143

[cpp2726-bib-0079] Rufino, K. A. , & Ellis, T. E. (2018). Contributions of cognitions, psychological flexibility, and therapeutic alliance to suicidal ideation in psychiatric inpatients. Suicide and Life‐threatening Behavior, 48(3), 271–280. 10.1111/sltb.12353 28485527

[cpp2726-bib-0080] Ryberg, W. , Diep, L. M. , Landrø, N. I. , & Fosse, R. (2019). Effects of the collaborative assessment and management of suicidality (CAMS) model: A secondary analysis of moderation and influencing factors. Archives of Suicide Research, 24(4), 589–608. 10.1080/13811118.2019.1650143 31442105

[cpp2726-bib-0081] Safran, J. D. , McMain, S. , Crocker, P. , & Murray, P. (1990). Therapeutic alliance rupture as a therapy event for empirical investigation. Psychotherapy, 27(2), 154–165. 10.1037/0033-3204.27.2.154

[cpp2726-bib-0082] Safran, J. D. , & Muran, J. C. (2000). Resolving therapeutic alliance ruptures: Diversity and integration. Journal of Clinical Psychology, 56, 233–243. 10.1002/(SICI)1097-4679(200002)56:2<233::AID-JCLP9>3.0.CO;2-3 10718606

[cpp2726-bib-0083] Safran, J. D. , & Muran, J. C. (2006). Has the concept of the therapeutic alliance outlived its usefulness? Psychotherapy, 43, 286–291. 10.1037/0033-3204.43.3.286 22122099

[cpp2726-bib-0084] Schneider, B. , Grebner, K. , Schnabel, A. , Hampel, H. , Georgi, K. , & Seidler, A. (2011). Impact of employment status and work‐related factors on risk of completed suicide. A case‐control psychological autopsy study. Psychiatry Research, 190(2–3), 265–270. 10.1016/j.psychres.2011.07.037 21890214

[cpp2726-bib-0085] Sharf, J. , Primavera, L. H. , & Diener, M. J. (2010). Dropout and therapeutic alliance: A meta‐analysis of adult individual psychotherapy. Psychotherapy, 47(4), 637–645. 10.1037/a0021175 21198249

[cpp2726-bib-0086] Shattock, L. , Berry, K. , Degnan, A. , & Edge, D. (2018). Therapeutic alliance in psychological therapy for people with schizophrenia and related psychoses: A systematic review. Clinical Psychology and Psychotherapy, 25(1), e60–e85. 10.1002/cpp.2135 28961352

[cpp2726-bib-0087] Shearin, E. N. , & Linehan, M. M. (1992). Patient‐therapist ratings and relationship to progress in dialectical behavior therapy for borderline personality disorder. Behavior Therapy, 23(4), 730–741. 10.1016/S0005-7894(05)80232-1

[cpp2726-bib-0088] Smith, A. R. , Zuromski, K. L. , & Dodd, D. R. (2018). Eating disorders and suicidality: What we know, what we don't know, and suggestions for future research. Current Opinion in Psychology, 22, 63–67. 10.1016/j.copsyc.2017.08.023 28846874

[cpp2726-bib-0138] Steketee, G. , Perry, J. C. , Goisman, R. M. , Warshaw, M. G. , Massion, A. O. , Peterson, L. G. , Langford, L. , Weinshenker, N. , Farreras, I. G. , & Kelller, M. B. (1997). The psychosocial treatments interview for anxiety disorders: A method for assessing psychotherapeutic procedures in anxiety disorders. Journal of Psychotherapy Practice and Research, 6(3), 194–210. 9185065 PMC3330464

[cpp2726-bib-0089] Sterne, J. A. C. , Egger, M. , Moher, D. , & Boutron, I. (2017). Addressing reporting biases. In J. P. T. Higgins , R. Churchill , J. Chandler , & M. S. Cumpston (Eds.), Cochrane handbook for systematic reviews of interventions (5.2.0) (pp. 1–49). Cochrane.

[cpp2726-bib-0090] Stiles, W. B. , Glick, M. J. , Osatuke, K. , Hardy, G. E. , Shapiro, D. A. , Agnew‐Davies, R. , Rees, A. , & Barkham, M. (2004). Patterns of alliance development and the rupture‐repair hypothesis: Are productive relationships U‐shaped or V‐shaped? Journal of Counseling Psychology, 51(1), 81–92. 10.1037/0022-0167.51.1.81

[cpp2726-bib-0091] Stratton, N. , Alvarez, M. M. , Labrish, C. , Barnhart, R. , & McMain, S. (2020). Predictors of dropout from a 20‐week dialectical behavior therapy skills group for suicidal behaviors and borderline personality disorder. Journal of Personality Disorders, 34(2), 216–230. 10.1521/pedi_2018_32_391 30179573

[cpp2726-bib-0092] Strunk, D. R. , Brotman, M. A. , & DeRubeis, R. J. (2010). The process of change in cognitive therapy for depression: Predictors of early inter‐session symptom gains. Behaviour Research and Therapy, 48(7), 599–606. 10.1016/j.brat.2010.03.011 20362978 PMC2878902

[cpp2726-bib-0093] Tarrier, N. , Gooding, P. , Pratt, D. , Kelly, J. , Awenat, Y. , & Maxwell, J. (2013). Cognitive behavioral prevention of suicide in psychosis. Routledge.

[cpp2726-bib-0094] Tarrier, N. , Taylor, K. , & Gooding, P. (2008). Cognitive‐behavioral interventions to reduce suicide behavior: A systematic review and meta‐analysis. Behavior Modification, 32(1), 77–108. 10.1177/0145445507304728 18096973

[cpp2726-bib-0095] Taylor, P. J. , Gooding, P. A. , Wood, A. M. , Johnson, J. , Pratt, D. , & Tarrier, N. (2010). Defeat and entrapment in schizophrenia: The relationship with suicidal ideation and positive psychotic symptoms. Psychiatry Research, 178(2), 244–248. 10.1016/j.psychres.2009.10.015 20472304

[cpp2726-bib-0113] Tichenor, V. , & Hill, C. E. (1989). A comparison of six measures of working alliance. Psychotherapy: Theory, Research, Practice, Training, 26(2), 195–199. 10.1037/h0085419

[cpp2726-bib-0110] Tracey, T. J. , & Kokotovic, A. M. (1989). Factor structure of the working alliance inventory. Psychological Assessment, 1(3), 207–210. 10.1037/1040-3590.1.3.207

[cpp2726-bib-0096] Tsai, M. , Ogrodniczuk, J. S. , Sochting, I. , & Mirmiran, J. (2014). Forecasting success: Patients' expectations for improvement and their relations to baseline, process and outcome variables in group cognitive‐behavioral therapy for depression. Clinical Psychology & Psychotherapy, 21(2), 97–107. 10.1002/cpp.1831 23280955

[cpp2726-bib-0097] Turner, R. M. (2000). Naturalistic evaluation of dialectical behavior therapy‐oriented treatment for borderline personality disorder. Cognitive and Behavioral Practice, 7(4), 413–419. 10.1016/S1077-7229(00)80052-8

[cpp2726-bib-0098] Vasquez, M. J. T. (2007). Cultural difference and the therapeutic alliance: An evidence‐based analysis. American Psychologist, 62(8), 878–885. 10.1037/0003-066X.62.8.878 18020774

[cpp2726-bib-0099] Weinberg, I. , Ronningstam, E. , Goldblatt, M. J. , & Maltsberger, J. T. (2011). Vicissitudes of the therapeutic alliance with suicidal patients: A psychoanalytic perspective. In K. Michel & D. A. Jobes (Eds.), Building a therapeutic alliance with the suicidal patient (pp. 293–316). American Psychological Association.

[cpp2726-bib-0100] Williams, J. M. G. (1997). Cry of pain: Understanding suicide and self‐harm. Penguin Books.

[cpp2726-bib-0117] Wilmers, F. , Munder, T. , Leonhart, R. , Herzog, T. , Plassmann, R. , Barth, J. , & Linster, H. W. (2008). The German version of the Working Alliance Inventory ‐ short revised (WAI‐SR) ‐ a cross‐school, economical and empirically validated instrument for recording the therapeutic alliance. Clinical Diagnostics and Evaluation, 1(3), 343–358.

[cpp2726-bib-0101] Windfuhr, K. , & Kapur, N. (2011). Suicide and mental illness: A clinical review of 15 years findings from the UK national confidential inquiry into suicide. British Medical Bulletin, 100(1), 101–121. 10.1093/bmb/ldr042 21948337

[cpp2726-bib-0102] Winter, D. , Bradshaw, S. , Bunn, F. , & Wellsted, D. (2014). A systematic review of the literature on counselling and psychotherapy for the prevention of suicide: 2. Qualitative studies. Counselling and Psychotherapy Research, 14(1), 64–79. 10.1080/14733145.2012.737004

[cpp2726-bib-0103] Wintersteen, M. B. , Mensinger, J. L. , & Diamond, G. S. (2005). Do gender and racial differences between patient and therapist affect therapeutic alliance and treatment retention in adolescents? Professional Psychology: Research and Practice, 36(4), 400–408. 10.1037/0735-7028.36.4.400

[cpp2726-bib-0104] World Health Organization . (2002). Handbook for good clinical research practice (GCP) guidance for implementation. World Health Organization.

[cpp2726-bib-0105] Zilcha‐Mano, S. (2017). Is the alliance really therapeutic? Revisiting this question in light of recent methodological advances. American Psychologist, 72(4), 311–325. 10.1037/a0040435 28481579

[cpp2726-bib-0106] Zilcha‐Mano, S. , Dinger, U. , McCarthy, K. S. , & Barber, J. P. (2014). Does alliance predict symptoms throughout treatment, or is it the other way around? Journal of Consulting and Clinical Psychology, 82(6), 931–935. 10.1037/a0035141 24274627 PMC4032804

